# The Outbreak of SARS-CoV-2 Pandemic and the Well-Being of Polish Students: The Risk Factors of the Emotional Distress during COVID-19 Lockdown

**DOI:** 10.3390/jcm10050944

**Published:** 2021-02-28

**Authors:** Dariusz Juchnowicz, Jacek Baj, Alicja Forma, Kaja Karakuła, Ryszard Sitarz, Jacek Bogucki, Hanna Karakula-Juchnowicz

**Affiliations:** 1 Department of Psychiatric Nursing, Medical University of Lublin, 20-093 Lublin, Poland; juchnowiczdariusz@wp.pl; 2 Chair and Department of Anatomy, Medical University of Lublin, 20-090 Lublin, Poland; jacek.baj@umlub.pl; 3 Student Research Group Department of Forensic Medicine, Medical University of Lublin, 20-090 Lublin, Poland; aforma@onet.pl; 4 Psychiatry Student Research Group I Department of Psychiatry, Psychotherapy and Early Intervention, Medical University of Lublin, 20-439 Lublin, Poland; kaja.karakula@gmail.com; 5 Chair and I Department of Psychiatry, Psychotherapy, and Early Intervention, Medical University of Lublin, 20-439 Lublin, Poland; hanna.karakula-juchnowicz@umlub.pl; 6 Chair and Department of Organic Chemistry, Faculty of Pharmacy Medical University of Lublin, 20-093 Lublin, Poland; jacekbogucki@umlub.pl

**Keywords:** COVID-19, SARS-CoV-2, pandemic, psychological well-being, DASS-21, depression, anxiety, stress, emotional distress

## Abstract

The coronavirus disease 2019 (COVID-19) pandemic has a significant impact on both physical and mental health. The aim of this cross-sectional study was to (1) evaluate depression, anxiety, and stress levels among students from Polish universities during the first weeks of the COVID-19 pandemic and (2) assess the risk factors of the higher intensity of emotional distress. We conducted an online survey using the Depression, Anxiety, and Stress Scale-21 (DASS-21) to assess well-being. The study included 2172 respondents (73% female, 27% male) with a mean age of 22.1 ± 2.2. Moderate to extremely severe scores of depression, anxiety, and stress were reported by 43.4%, 27.3%, and 41.0% of the respondents, respectively. Higher scores of DASS-21 were related to female sex (odds ratio (OR) = 3.01), studying sciences (OR = 2.04), co-residence with the roommates (OR = 1.25), suffering from a mental disorder (OR = 5.88), loneliness (OR = 293.30), the usage of psychiatric support before pandemic (OR = 8.06), poor economic situation (OR = 13.49), and the lower scores were found for being currently employed (OR = 0.4). This study highlights an urgent need for (1) crisis-oriented psychological and psychiatric support for students during the outbreak of the COVID-19 pandemic and (2) preparing appropriate psychological interventions to improve the mental health of students for a possible similar situation in the future.

## 1. Introduction

A new viral disease due to the infection by the severe acute respiratory syndrome coronavirus-2 (SARS-CoV-2) has been named by the World Health Organization (WHO) as the coronavirus disease 2019 (COVID-19) and announced as a pandemic approximately five months after the 41 first reported cases of pneumonia in Wuhan, China [1,2,3]. Before it was officially reported as a pandemic, the spread of the SARS-CoV-2 has affected 114 countries, leading to nearly 188,000 infections, among which thousands of them included critical cases, as well as over 4000 deaths [4,5]. According to the most recent statistics, there are more than 100 million cases confirmed so far, and the number of new cases (confirmed or fatal) is continually increasing every other day.

Compared to the severe acute respiratory syndrome coronavirus (SARS-CoV) or the Middle East respiratory syndrome coronavirus (MERS-CoV), SARS-CoV-2 has affected a significantly greater number of people during the outbreak of the pandemic, mainly due to its higher transmission potential [6] and its efficient spread by various transmission routes including airborne, contact, or fecal-oral routes [7,8]. Several factors such as gender, age, or the presence of concomitant diseases have and an impact on the severity of SARS-CoV-2 infection that might range from the asymptomatic infection to the clinical conditions characterized by severe respiratory failure or death [5,8]. Factors that contribute to the higher risk of mortality rates of the infected patients include cardiovascular diseases, hypertension, respiratory diseases, diabetes, older age, obesity [6,8], or vitamin D deficiency [9].

According to the WHO, on 26 April 2020, which was the closing day of our survey, the course of events proceeded as follows—2,804,796 confirmed cases, 193,710 confirmed deaths [10]. From the date of the outbreak, in order to prevent the spread of the virus, many governments ordered the first recommendations regarding national lockdowns as well as traveling restrictions [11]. In Poland, on the same date (26 April 2020), there were 11,617 officially confirmed cases and 535 deaths already reported [12]. Numerous restrictions introduced by the governmental measures and public health recommendations during the COVID-19 pandemic have affected the daily living of the society, and those that primarily mattered were social distancing, social isolation, and home confinement. On 23 March, the Polish government announced a state of emergency due to the SARS-CoV-2 pandemic, and all schools and universities were closed [13]. From the 1 April till 26 April, people were not allowed to go out except in special cases such as work and going to the pharmacy, hospital, and grocery shops, besides, strict restrictions regarding the total number of customers who were allowed to be in the same room were introduced by the government. Moreover, the hours between 10 am and 12 am have been declared as ‘the hours for seniors only’ in all of the open institutions; parks, forests, and boulevards have been closed for all other citizens. Juveniles under 18 years old were not allowed to walk unsupervised by an adult [14]. People started to self-isolate, equipped with excess food, protective masks, and disinfectants. Incidences of shortages of masks and health equipment in numerous hospitals and pharmacies occurred [15]. Throughout the pandemic, information on the transmission dynamics, incubation time, basic reproductive frequency of COVID-19, or symptoms and clinical manifestations of the infection were unclear and continually changing. The absence of a specific cure or vaccine made the public more concerned about their health [16].

The risk of infection or death was not the only problem that was concerning society. Reports on the expansion of SARS-CoV-2 infection and new incidents of confirmed or fatal COVID-19 cases were more likely to be causing fear, anxiety, and distress [17]. The rapid spread of the virus, social isolation, changing of the command habits, many restrictions, postponing exams, reopening of schools and universities are pressuring the mental health of societies [10]. Moreover, the stigmatization or even discrimination of the individuals who might be associated with the area of spread of disease (e.g., healthcare workers) might be even more distressing [18]. In 2002, during the SARS pandemic, studies showed that the psychological impact on the non-infected community was higher in the younger population; besides, they presented with an increased self-blame, which could reflect frustration and guilt related to responsibility attribution [19]. There is an increasing number of studies that aim to assess the mental health of the general population during the COVID-19 pandemic; however, the long-term consequences remain questionable [20].

## 2. Psychological Consequences of the Pandemic

The outbreak of the SARS-CoV-2 pandemic has become an extremely pronounced stressor, which has extended to almost all of the countries worldwide. The fast spread of the pandemic was observed to affect all groups in society. At the current state of knowledge, it is believed that not only the pandemic alone but also the regulations and political measures that aim to prevent the spread of the virus have a significant impact on the mental health of societies. Researchers addressed the psychological and behavioral responses of people during the early stage of the COVID-19 pandemic and noticed higher depressive, anxiety, and stress symptoms in the general population [15,16,18,21,22,23,24]. Some researchers observed that particularly students’ psychological health was more influenced by the pandemic crisis compared to the other groups but presented with similar stress and anxiety levels [15]. There is some evidence that youths have reported depressive symptoms at a higher prevalence than the older ones [23]. A systematic review by Xiong et al. showed that the major risk factors associated with mental distress during the COVID-19 pandemic primarily included the student status, female gender, age groups < 40 years old, unemployment status, as well as the presence of either psychiatric or other chronic diseases [25]. In a study that combined the population of 113,285 individuals, it was demonstrated that the prevalence rate and intensity of depression, anxiety, and stress were significantly higher during the pandemic; besides, sleep disturbances and more intense psychological distress were also observed in a studied population [26]. During the first weeks of the pandemic outbreak, about half of the Chinese respondents’ reported a moderate-to-severe psychological impact, whereas one-third of the studied group—moderate-to-severe anxiety [15]. Similar to the above-mentioned studies, student status and female gender were associated with greater levels of depression, anxiety, and stress. In the general population of Austria, depressive and anxiety symptoms were up to five and three times more prevalent, respectively, compared to the results obtained before the outbreak of the pandemic [27]. Unemployment, financial instability, and a lower income, in general, are major economic factors that might exaggerate both psychological and mental consequences during a pandemic [28,29,30]. Even though the outbreak began in China, a study comparing the distress between Poles and Chinese showed that Polish society tends to present significantly higher depression, anxiety, and stress levels [31]. Generally, younger individuals are more susceptible to depressive, anxiety, and stress symptoms related to the pandemic, comparing to the older population [32]. According to the most recent studies, the pandemic seems to affect individuals in all age groups. Several factors such as student status or female gender tend to additionally exaggerate psychological distress; however, there are also protective factors such as social support, proper relationships with family and friends, or the absence of any mental illnesses [33]. A study by Epifanio et al. performed in Italy during COVID-19 lockdown showed that younger adults (18–34 years old) presented with the lowest levels of psychological health, constituting the most vulnerable subjects in the general population at the same time [34]. Since numerous studies have already reported that students are most vulnerable to emotional distress during the pandemic, it is of great importance to find the risk and protective factors associated with the emotional distress in this group [35,36,37,38,39].

Therefore, the aim of this study was to (1) evaluate depression, anxiety, and stress levels among students from Polish universities during the first weeks of the COVID-19 pandemic, (2) assess the risk factors that increase the probability of the higher intensity of emotional distress, (3) create a portrait of a student who requires enhanced emotional support during the pandemic, (4) compare the results of our study with the results of research from other countries, that have also assessed the emotional well-being of the students during the first stages of COVID-19 lockdown with the usage of the same psychological tools.

## 3. Materials and Methods

### 3.1. Study Design and Survey Description

Before the survey preparation, the authors performed an independent review of the literature regarding the impact of the COVID-19 pandemic on mental health with a particular emphasis on the students. Subsequently, a structured questionnaire was created, including four parts: (1) sociodemographic data, (2) questions related to one’s health condition, (3) economic situation, and (4) Polish adaptation of the Depression, Anxiety, and Stress Scale-21 (DASS-21) to assess depression, anxiety, and stress levels [40]. An anonymous online cross-sectional survey included the questionnaire that was distributed on 20 April 2020 via social media among Polish students from 87 universities, including all medical universities in Poland. The survey was prepared via a Google form and was posted on social media groups on Facebook that gather students from Poland. The questionnaire was also sent via e-mail to other universities in Poland with a request to distribute it to the private groups at universities as well. The survey was closed on 26 April. Therefore, it was conducted almost 6 weeks after applying the lockdown measures in Poland on 10 March. Hereby, the snowball sampling method was utilized. The respondents were completing the survey individually in an estimated average time of about 10 min. All the answers given by the respondents were confidential, and only those who were conducting the research had access to the answers.

### 3.2. Measures

The questionnaire was composed in such a way as to provide the most crucial information regarding the respondent’s sociodemographic data, economic situation, and general, subjective knowledge about COVID-19. Sociodemographic data included (1) gender, (2) age, (3) field of study, (4) year of study, (5) place of residence, (6) place of residence during the COVID-19 pandemic, (7) marital status, (8) parental status, and (9) living situation and co-residence. Data related to student health conditions included questions regarding whether someone got COVID-19, cases of COVID-19 among family members and/or friends, deaths due to COVID-19 among family members and/or friends, active involvement in the fight against SARS-CoV-2, using the support of a psychologist/psychiatrist before the pandemic, taking medications or supplements that improve the immunity, and individual difficulties related to the current epidemiological situation. The section related to the economic situation included the following questions—(1) whether a respondent is currently working and (2) how the respondent assesses their economic situation during the pandemic.

### 3.3. DASS-21 Scale

The mental health status of the respondents was measured using the Depression, Anxiety, and Stress Scale-21 Items (DASS-21) [40]. In the following study, a polish adaptation of the DASS-21 scale was used [41]. The DASS-21 is a shortened version of the original 42-item DASS created by Lovibond and Lovibond, and both of them are self-report scales designed to estimate the overall emotional distress of a respondent, as well as to assess and evaluate the scores of the depression, anxiety, and stress levels [42,43]. It has been proved that both of the DASS scales show a high internal consistency [44]. The DASS-21 is composed of a hierarchical factor structure that includes the three first-order factors (depression, anxiety, and stress), as well as one second-order factor (emotional symptoms) [45]. Such a designated scale is suitable for both clinical and non-clinical purposes. An advantage is that compared to DASS-42, a shortened version (DASS-21) requires less time to be fulfilled by the respondents, providing similar outcomes at the same time. The main difference between these scales is the fact that the DASS-42 scale is preferably chosen for clinical purposes, whereas the DASS-21 is primarily chosen for research purposes. To compare the results with the normative data and scientific publications in which the DASS-42 scale was used, the statistical results obtained from the DASS-21 should be multiplied by 2. Such a conversion provides the possibility to obtain the validity of the statistical results that are comparable to those that are obtained while applying the DASS-42, however, being less time-consuming and more legible for the respondents at the same time [46].

The DASS-21 scale consists of three major scales (depression, anxiety, and stress), among which, each of them contains 7 items. The depression scale evaluates the lack of interest, devaluation of life, hopelessness, dysphoria, anhedonia, inertia, and self-deprecation. The anxiety scale evaluates the general autonomic agitation, situational anxiety, and a subjective experience of anxiety, whereas the stress scale assesses the chronic non-specific arousal such as tension, irritability, and nervousness. Responses were structured by a 4-point Likert scale ranging from 0 (‘does not apply to me at all’) to 3 (‘applies to me very much or most of the time’), with higher scores indicating more negative experience in the past week. The total score of the DASS-21 ranges from 0 to 63, whereas the score for each of the subscales ranges from 0 to 21. After multiplication by 2 for further analysis of the results, we obtained a DASS-21 total maximum score of 126 and scores for every subscale equal to 42. The division of the total score multiplied by 2 of the depression, anxiety, and stress subscale is presented in Table 1 [42].

In our study, the alpha coefficients for the reliability of the depression, anxiety, stress, and full scale in the entire group were 0.95, 0.89, 0.96, and 0.94, respectively. The calculated values of Cronbach’s alpha for individual scales indicated the high reliability of the used scale.

### 3.4. Description of the Study Group

The final sample consisted of 2172 students of whom 73% (*n* = 1585) were women and 27% (*n* = 587) were men. The mean age of the sample was 22.1 ± 2.2. The majority of the respondents (60.5%) were medical students; other fields of study included students studying social sciences (19.2%), engineering (10%), arts and humanities (5%), and sciences (4.4%). The majority of students were in the first year of university (23.5%), and the least (4.9%), during the sixth year of university. The most prevalent place of residence of the students was the village (22.9%). In the studied group, most of the students were single (65.6%) or in an informal relationship (30.7%), whereas only 2.5% of the studied group was married. Regarding the place of residence during the COVID-19 pandemic, 48.3% of the respondents have answered that they are currently living with their parents. The smallest number of students answered that they lived with a partner and a child 1% (*n* = 21). The sociodemographic data of the respondents is presented in Table 2.

### 3.5. Characteristic of the Respondents’ Health Status

The majority of the respondents (*n* = 2112; 97.2%) and their relatives and friends (*n* = 1943; 89.5%) did not suffer from COVID-19. Only 1.3% of the students, as well as 2.4% and 7.4% of students, either a family member or friend respectively, had gotten COVID-19. In this group, 0.6% of students suffered from a loss of a family member or friend due to COVID-19. Some students (20.2%) took part in an active fight against the spread of SARS-CoV-2 by sewing protective masks, helping elderly people with shopping, collecting money for hospital equipment, providing telephone consultations, and volunteering in hospitals. The majority of respondents (82.9%) did not get any psychological or psychiatric support before the outbreak of the pandemic, which led to the conclusion that 17.1% of the respondents had experienced some kind of mental disorder before. This data is consistent with the global data on the prevalence of mental disorders—about 17.6% of the population meets the criteria for common mental disorders [47]. With regards to the students who used such support, the psychological ones were most frequently chosen by the students (*n* = 162; 7.5%). The majority of the respondents (*n* = 1506; 69.3%) did not take any supplements or medicines to improve their immunity. From those who took supplements (*n* = 665; 30.6%), in most of the cases it was vitamin D (*n* = 157; 7.2%), vitamin C (*n* = 140; 6.5%), or a vitamin complex (*n* = 134; 6.2%), and the rest used other medications (*n* = 234; 10.8%), including magnesium, omega-3 fatty acids, herbs, and homeopathic remedies. Regarding the difficulties that were faced by the students during the pandemic, most of the students (*n* = 728; 33.5%) were afraid of infecting their relatives. Interestingly, the fear of being infected by oneself was the least prevalent fear amongst students (*n* = 66; 3.0%). In the case of chronic diseases, the majority of students denied the existence of any (*n* = 1837; 84.6%). However, 4.1% (*n* = 88) suffered from thyroid diseases, 2.5% (*n* = 54) from asthma, 1.9% (*n* = 41) from a mental disorder, 1.5% (*n* = 32) from an allergy, 0.7 (*n* = 15) from diabetes, and 4.8% (*n* = 105) from other diseases. The data of respondents’ health status is provided in Table 3.

### 3.6. Employment Status and Economic Situation of the Respondents

We asked the students if they were working before the pandemic broke out. Most of the respondents (*n* = 1469; 67.6%), answered that they did not work, whereas the smallest group of students (*n* = 30; 1.2%) answered that they ran their own business. During the pandemic, 15.2% of the students lost their job, which caused to rise in the unemployment group of students to 82.8% (*n* = 1799). We also asked how the students assessed their economic situation during the pandemic. Most of the respondents (*n* = 1278; 58.8%) answered that they have a stable family income, and nothing has changed for them. The smallest number of students (*n* = 21; 1%) answered that they had to start borrowing money from family or friends during the outbreak of the pandemic because they were not able to support themselves. The data related to respondents’ economic situation is provided in Table 4.

## 4. Statistical Analysis

The statistical analysis of the results obtained in this study included descriptive statistics, distribution of the numbers with a percentage distribution, nonparametric tests (since data distribution obtained in a DASS test deviated from the normal distribution), U Mann–Whitney, as well as H Kruskal–Wallis tests with multiple comparisons test with Bonferroni correction. The distribution of the psychological variables was checked with the usage of analysis of histograms and the Shapiro–Wilk test in the Statistica program. The distribution in particular scales and the overall result deviated from the normal distribution, which was statistically significant. The effect size for the Kruskal–Wallis test was calculated as the eta squared based on the H-statistic: eta squared (H) = (H − k + 1)/(*n* − k); where H is the value obtained in the Kruskal–Wallis test; k is the number of groups; *n* is the total number of observations. To check the reliability of the applied test, the values of the Cronbach’s alpha test coefficient for individual scales of the tool were calculated (using the Statistica v.13 program; Statistica software—Polish version from StatSoft Corporation Poland, the partner of Tibco Corporation, Palo Alto, California, USA (licence for Medical University of Lublin)). To determine the risk factors for developing higher intensity of emotional distress, we standardized the scores of the DASS-21 total score, and then we distinguished two groups of subjects for further comparisons. The first group of subjects included the respondents with low scores (DASS 0; below mean (M)—standard deviation (SD)), whereas the second group—those with high scores (above M + SD). Afterward, odds ratio (OR) values along with the significance level were calculated using the MedCalc Odds Ratio Calculator program.

The control groups were allocated to the particular variable as follows—for ‘sex’—men in the DASS 0 and DASS 2 groups; for ‘field of study’ and ‘I live with’—the whole DASS 0 and DASS 2 groups; for ‘Do you have any chronic disease’—the respondents who answered ‘No’ in the DASS 0 and DASS 2 groups; for ‘Did you use psychological/psychiatric help before the beginning of the pandemics?’ and ‘Did you use psychological/psychiatric help during the pandemics’—the respondents who answered ‘No’ in the DASS 0 and DASS 2 groups; for ‘What was the most difficult for you during pandemics?’—the respondents who answered ‘I was not afraid’ in the DASS 0 and DASS 2 groups; for ‘Are you currently working’—the respondents who answered ‘No, I do not work’ in the DASS 0 and DASS 2 groups; for ‘How do you assess your economic situation during pandemics?’—the respondents who answered ‘I have a stable family income, nothing has changed’ in the DASS 0 and DASS 2 groups.

## 5. Ethical Considerations

This study was approved by the Bioethical Commission of the Medical University of Lublin, Lublin, Poland, and was conducted in compliance with national legislation and the Declaration of Helsinki. Before fulfilling the questionnaire, all of the respondents had to sign a consent form declaring that they agree to take part in this study before the survey has started. Informed consent included information about the form and nature of the study along with its aim and information about the confidentiality and anonymity of data and the exploitation of the results only for scientific purposes. All of the data obtained in this study was gathered and analyzed anonymously.

## 6. Results

### 6.1. DASS-21

#### 6.1.1. Total DASS Score

The total DASS score for the entire group of the respondents was 38.13 ± 26.51, which was lower than the cut-off score equal to 60, proposed by Lovibond and Lovibond [41]. The overall emotional distress was statistically higher (*p* < 0.001) in females (M = 40.54 ± 26.65) compared to males (M = 31.60 ± 25.02). The results showed that there was a relationship between the field of study and the DASS total score; the highest score was observed in science students (median (Me) = 42.00 ± 40.91), the second in turn was in the case of the participants studying arts and humanities (Me = 39.00 ± 28.98), social sciences (Me = 35.00 ± 28.14), and engineering (Me = 32.00 ± 26.79). The lowest score was obtained amongst the respondents who studied medicine (Me = 31.00 ± 25.48). Based on the Kruskal–Wallis test, the studied groups were statistically different (H = 16.16, *p* = 0.0028). The highest difference was noted between the medical and science students (z = 3.312, *p* = 0.009) (Supplementary Table S1). There were no statistical differences between the first-year students and the rest of the respondents in the DASS total score, as well as in none of the subscales (depression, anxiety, and stress).

#### 6.1.2. Depression

The mean score for the depression subscale for the entire study group was 14.04 ± 10.44, which can be classified as a moderate level of depressive symptoms. The intensity of depression defined as normal in the DASS-21 subscale concerned 43.6% (*n* = 948) of the total group of students. The mild intensity of depression applied to 13% (*n* = 282) of the examined students, moderate to 19.9% (*n* = 432), severe to 10.2% (*n* = 221), and extremely severe to 13.3% (*n* = 289). The level of depression in the female group (M = 18.41 ± 14.05) was significantly higher (*p* < 0.001) than in males (M = 12.24 ± 14.37). The results obtained from the Kruskal–Wallis test showed no significant difference between the students’ field of study and the level of depression (Supplementary Table S1.2).

#### 6.1.3. Anxiety

In the anxiety subscale of DASS-21, the mean result for the entire group was 7.71 ± 8.29 (equivalent for mild anxiety). Among the examined students, the intensity of anxiety defined as normal in the DASS-21 subscale concerned 60.2% (*n* = 1307) of the respondents. Mild anxiety affected 12.5% (*n* = 273) of the respondents, moderate—9% (*n* = 195), severe—6.6% (*n* = 144), extremely severe—11.7% (*n* = 253). Again, as in the depression subscale women (M = 13.19 ± 11.54) reached (*p* < 0.001) a higher level of anxiety than male participants (M = 6.90 ± 9.92). Students of arts and humanities (Me = 6.00 ± 9.63), science (Me = 6.00 ± 9.2), social sciences (Me = 6.00 ± 8.63) reached the highest score, whereas medicine (Me = 4.00 ± 8.0) and engineering (Me = 4.00 ± 8.06) the lowest. The results did not vary significantly between different fields of studies (Supplementary Table S1.3).

#### 6.1.4. Stress

The *mean* value for stress *intensity* for the whole group was 16.93 ± 10.98, which classifies as a mild level of stress. The stress level among the surveyed students, which is defined as normal in the DASS-21 subscale, concerned 47.2% (*n* = 1026) of the respondents, mild stress—11.8% (*n* = 255), moderate stress—15.3% (*n* = 333), severe stress—16.8% (*n* = 364), and the extremely severe stress—8.9% (*n* = 194) of the respondents. As previously, females (20.93 ± 15.45) scored statistically (*p* < 0.001) higher than males (12.58 ± 14.70). With regards to the anxiety subscale, the order was almost the same as in the case of the above-mentioned subgroups with science faculty (Me = 20.00 ± 11.12) in the highest place, then art and humanities (Me = 19.00 ± 11.62), and faculty of social sciences (Me = 16.00 ± 11.51), engineering (Me = 16.00 ± 10.82), and medicine (Me = 16.00 ± 10.73) with the lowest scores. The Kruskal–Wallis test showed no difference between the results of the stress subscale and the field of study (Supplementary Table S1.4).

The number of students with a particular degree of severity of depression, anxiety, and stress levels is presented in Table 5. Most of the students reached a normal level of depression (*n* = 948, 43.6%), anxiety (*n* = 1307, 60.2%), and stress (*n* = 1026, 47.2%). The highest level of depression symptoms was noted in 13.3% (*n* = 289) of the respondents. In the case of anxiety and stress, 11.7% (*n* = 253) and 8.9% (*n* = 194) of the students, respectively, were observed to have extremely severe symptoms (Table 5).

### 6.2. Factors Correlating with the Emotional Distress in the Study Group

#### 6.2.1. Demographic Factors

One of the main goals of our study was to identify the factors that increase the probability of a higher intensity of emotional distress in the group of students (Supplementary Table S8).

Among the demographic factors associated with an increased intensity of the emotional response in the study group were female sex (OR = 3.01, 95% CI: 2.15–4.22) and science as a field of study (OR = 2.04, 95% CI: 0.99–4.19).

#### 6.2.2. The Most Difficult Problem during the Pandemic

The respondents were asked to choose from the nine situations the only one that in the moment of the outbreak of the pandemic and through the first weeks was the most difficult for them. Students who choose ‘loneliness’ as the most difficult were observed to reach the highest level of overall emotional distress (Me = 52.00 ± 26.01), depression (Me = 20.00 ± 11.33), anxiety (Me = 8.00 ± 8.37), and stress (Me = 22.00 ± 10.41) measured by the DASS-21. Taking into consideration the intensity of the total DASS scores, the respondents who chose ‘radical change in style and way of life’ (Me = 35.00 ± 28.43) as the second one in ranking and the third as ‘isolation’ (Me = 34.00 ± 27.53) ex aequo with ‘financial instability’ (Me = 34.00 ± 27.64). In case of level of depression and stress, second were ‘radical change in style and way of life’ (depression: Me = 12.00 ± 11.27, stress: Me = 18.00 ±11.25) and ‘isolation’ (depression: Me = 12.00 ± 10.24, stress: Me = 18.00 ± 11.33). With regards to the anxiety subscale, the ‘fear of their own risk of infection illness, death’ (Me = 6.00 ± 10.09), ‘fear of infection of the loved ones’ (Me = 6.00 ± 7.67), and “financial instability’ (Me = 6.00 ± 8.31) were associated with the highest levels of anxiety. In total DASS and all subscales the differences between the groups were statistically significant—DASS-21 (H = 87.51, *p* < 0.001), depression (H = 106.68, *p* < 0.001), anxiety (H = 53.83, *p* < 0.001), stress (H = 74.58, *p* < 0.001) (Supplementary Tables S2, S2.2, S2.3, and S2.4). The increased feeling of loneliness was related to 292-fold higher odds of developing higher overall emotional distress (OR = 292, 95% CI: 15.77–5454.92).

#### 6.2.3. Suffering from Chronic Diseases

In the case of the concomitant chronic diseases, the highest scores of overall emotional distress (Me = 58.00 ± 29.26), depression (Me = 20.00 ± 11.25), stress (Me = 24.00 ± 11.67), and anxiety (Me = 12.00 ± 11.68) were observed in people with mental disorders. The suffering from any chronic diseases was associated with the occurrence of overall emotional disorders in DASS-21 above the cut-off score. The presence of psychiatric disorders was significantly associated with a higher level of overall emotional distress (z = 4.831, *p* < 0.001), depression, (z = 4.424, *p* < 0.001) anxiety (z = 4.148, *p* = 0.001), and stress (z = 4.142, *p* = 0.001), compared to the respondents without any chronic diseases (Supplementary Tables S3, S3.2, S3.3, and S3.4). Students suffering from psychiatric disorders were almost six times more likely to exaggerate their emotional responses than those who had no mental health problems (OR = 5.89, 95% CI: 1.70–20.27). The usage of any supplements or medication to improve subject immunity did not correlate with the results of the DASS-21.

#### 6.2.4. Psychological/Psychiatric Support before the Pandemic Broke-Out

Students who were using psychological and/or psychiatric help had the highest results in overall emotional distress (Me = 56.00 ± 29.53), depression (Me = 20.00 ±10.43), anxiety (Me = 12.00 ±10.43), and stress (Me = 24.00 ± 11.61). The use of the psychological and/or psychiatric support before the pandemic was associated with significantly higher levels of all the assessed variables of the DASS-21 (overall emotional distress (H = 102.21, *p* < 0.001); depression (H = 78.208, *p* < 0.001); anxiety (H = 92.198 *p* < 0.001); stress (H = 76.923, *p* < 0.001), and the total DASS scores were above the cut-off scores (Supplementary Tables S4, S4.2, S4.3, and S4.4). The respondents who had higher odds of emotional distress to the pandemic situation were those who used psychiatrist services before the outbreak of the pandemic (OR = 8.06, 95% CI: 2.79–23.28).

#### 6.2.5. Economic Situation during a Pandemic

The self-assessment of students’ economic situation in the time of pandemic was significantly related to overall emotional distress (H = 63.77, *p* < 0.001), depression (H = 51.86, *p* < 0.001), anxiety (H = 58.79, *p* < 0.001), and stress (H = 51.44, *p* < 0.001). Students who gave the answer ‘stable family income, nothing has changed’ had the lowest result in overall emotional distress (Me = 30.00 ± 25.43), depression (Me = 10.00 ± 10.09), anxiety (Me = 4.00 ± 7.80), and stress (Me = 14.00 ± 10.71) (Supplementary Tables S5, S5.2, S5.3, and S5.4). More than 13-fold higher odds (OR = 13.49, 95% CI: 1.71–106.33) of an increased emotional response was observed in the respondents who chose the answer that they had to start borrowing money from family or friends during the outbreak of the pandemic because they did not have sufficient funds to support themselves.

#### 6.2.6. Employment Status during a Pandemic

The employment status during the pandemic was associated with the intensity of emotions measured with DASS-21—overall emotional distress (H = 17.76, *p* < 0.001) and depression (H = 30.49, *p* < 0.001). In total DASS scores and all subscales, the sequence was similar, reaching the highest levels in the group of students who were not working (overall emotional distress—Me = 34.00 ± 29.66, depression—Me = 12.00 ± 10.47, anxiety—Me = 6.00 ± 8.41, stress—Me = 16.00 ± 10.92), in later order—in a group of the respondents that were working mentally, physically, and lastly—running their own businesses. The level of overall emotional distress (Me = 34.00 ± 26.66) observed in students who were currently not working reached above the cut-off score (Supplementary Tables S6, S6.2, S6.3, and S6.4). Currently working mentally (OR = 0.5, 95% CI: 0.34–0.85) and physically (OR = 0.4, 95% CI: 0.18–1.05) were related to a 0.5 and 0.4-fold lower odds of increased overall emotional distress respectively.

#### 6.2.7. Living Situation

The living situation and the co-residence were observed to correlate with the results of the depression subscale (H = 37.22, *p* < 0.001). The highest levels of overall emotional distress (Me = 34.00 ± 25.82), depression (Me = 12.00 ± 10.32), anxiety (Me = 6.00 ± 8.18), and stress (Me = 16.00 ± 10.68) were found in a group of students living with roommates and students living with parents—overall emotional distress (Me = 34.00 ± 27.01), depression (Me = 12.00 ± 10.58), anxiety (Me = 6.00 ±8.42), and stress (Me = 16.00 ± 11.08). People living with roommates and those living with their parents reached above the cut-off score in overall emotional distress results. The students who live with roommates presented 1.25-fold higher odds of depression, stress, and anxiety (OR = 1.25, 95% CI: 0.89–1.78) (Supplementary Tables S7, S7.2, S7.3, and S7.4).

### 6.3. Comparison with the Results of Other Authors’ Studies

The results of our study have been compared with the ones conducted in Spain, China, India, and Bangladesh. The following countries were chosen by us since the other authors applied the same psychological instrument (DASS-21) and their studies were performed during the first stages of the pandemic, similarly to our research (Table 6).

Regarding the total DASS scores, higher emotional distress was noted among Polish students compared to Chinese students (*t* = 20.44, *d* = 0.76). Polish students also showed a greater severity of depression, anxiety, and stress in comparison to Spanish students (depression—*t* = 42.31, *d* = 1.04; anxiety—*t* = 27.40, *d* = 0.68; stress *t* = 10.12, *d* = 1.20). Statistically significant results were obtained between Poland and Bangladesh, where higher levels of depression, anxiety, and stress were shown by students from Bangladesh (depression—*t* = −11.35, *d* = 1.87; anxiety—*t* = −23.66, *d* = 1.07; stress *t* = −14.23, *d* = 1.91). Bangladeshi students also showed higher emotional intensity compared to Spanish students in all of the investigated subscales (depression—*t* = −60.44, *d* = 1.50; anxiety—*t* = −59.69, *d* = 0.69; stress *t* = −72.65, *d* = 1.40).

## 7. The Portrait of a Student who May Potentially Require Special Psychiatric and/or Psychological Support during the Pandemic

Based on the results of our study, the profile of a student who requires potential psychological or/and psychiatric support during the pandemic is a woman (OR = 3.01, 95% CI: 2.15–4.22), studying science (OR = 2.04, 95% CI: 0.99–4.19), living with her roommates (OR = 1.25, 95% CI: 0.89–1.78), suffering from mental disorders that appeared before the outbreak of the pandemic (OR = 5.88, 95% CI: 1.70–20.27), who was using psychiatric support before the outbreak of the pandemic (OR = 8.06, 95% CI: 2.79–23.28), complained of loneliness during the pandemic (OR = 293.31, 95% CI: 15.77–5454.92), and was in a difficult economic situation that forces her to borrow the money from the family or friends during the outbreak of the pandemic to support herself (OR = 13.49, 95% CI: 1.71–106.33).

## 8. Discussion

The rapid outbreak of the COVID-19 pandemic has forced many countries to introduce specific social distancing and lockdown measures. Such restrictions have a significant impact on the overall well-being and might develop and progress in the form of symptoms related to depression, anxiety, or stress [48]. The SARS-CoV-2 virus, as an unknown agent with undefined mortality and infectivity, undoubtedly had an impact on mental health. The contemporary world is not used to the situation that has arisen. Moreover, frequent media releases about new infection incidents and deaths could heighten the fear of the threat.

The main aim of this cross-sectional study, including a population of more than two thousand Polish students, was to assess depression, stress, and anxiety during the first weeks after the outbreak of the SARS-CoV-2 pandemic and the lockdown in Poland. We also searched for possible risk factors that may intensify students’ emotional responses. Results of our study showed that moderate to extremely severe scores of depression, anxiety, and stress were reported by 43.4%, 27.3%, and 41.0% of the respondents, respectively.

After processing the results of our research, we decided to compare the DASS-21 results received in our study with those obtained by researchers in the period before the COVID-19 pandemic. The study was conducted in the winter semester 2018/2019 at the Jagiellonian University among Polish medicine students showed that the level of overall emotional distress was lower than those in our study. Unexpectedly, we noticed a lower severity of anxiety in comparison to the results obtained before the pandemic in the above-mentioned study. However, the authors of the study did not present the percentage distribution of the results achieved, making further comparisons in this matter impossible [49]. In a study by Martinotti et al. using an Italian population, the depression rate was lower (22.9%), whereas anxiety was greater (30.1%) compared to the results of our study. Besides, the authors also showed irritability (31.6%) and post-traumatic stress symptoms (5.4%) as one of the most prevalent during the quarantine period [50]. What is intriguing and might be interesting for further research is that negative emotions (fear, anxiety, and sadness) experienced less intensely but not less frequently could constitute a protective role of trait emotional intelligence during the COVID-19 pandemic, according to the Polish study performed by Moroń and Biolik-Moroń [51].

**Figure 1:**
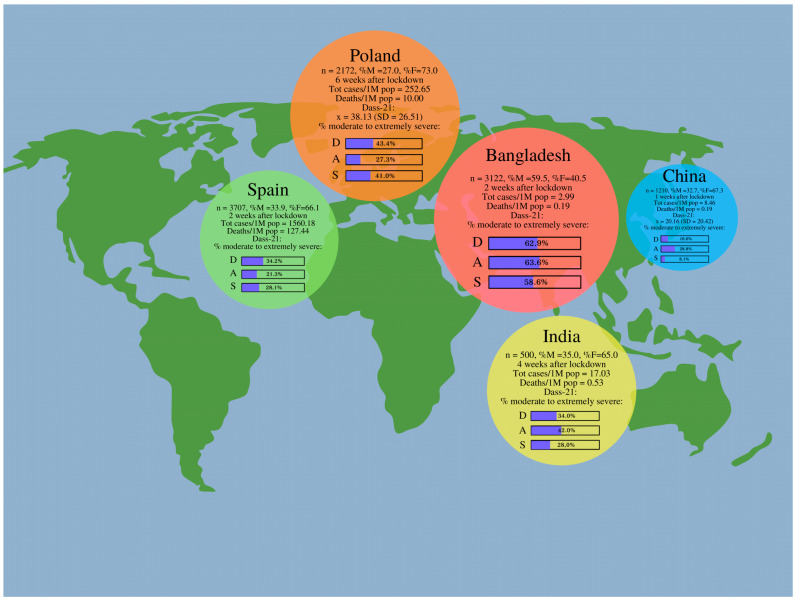
The results of the studies from Poland, Spain, China, India, and Bangladesh performed applying the DASS-21 scale. Legend: *n* = total number of the respondents, %M = percentage of male respondents, Tot cases/1M pop = total number of cases of COVID-19 per 1 million population on the first day of study, Tot deaths/1M pop = total number of deaths caused by COVID-19 per 1 million population on the first day of study, x = mean score of DASS-21, SD = standard deviation, % moderate to extremely severe = percentage of participants with Dass-21 score form moderate to extremely severe for depression (D), anxiety (A), and stress (S).

It seemed interesting to compare the intensity of the emotional distress presented by Polish students with the reaction of students from other countries during the coronavirus pandemic. We have reviewed the available literature and selected four countries from different parts of the world, including Spain, China, India, and Bangladesh, which also performed similar studies primarily on populations of students and used the DASS-21 scale (Figure 1).

Considering the time frames, China was the first to measure the intensity of the emotional response in the student population. The study in China was conducted three days after the WHO announced COVID-19 as a public health emergency [48]. Our study was conducted at the end of the lockdown in Poland, whereas the Chinese study—only after one week of the lockdown. Polish students presented significantly higher emotional distress compared to Chinese students. Compared to the other analyzed countries, China was the one with the lowest percentage of students with moderate to extremely severe depression. This result was consistent with Selye’s Theory of Stress—General Adaptation Syndrome, in which anxiety and stress dominate in the initial alarm reaction stage, and depression appears only in the third phase—exhaustion stage [52]. In a meta-analysis conducted by Salari et al., which aimed to investigate the rates of depression, anxiety, and stress during the COVID-19 pandemic by particular continents, it turned out that Asia presented the highest prevalence of anxiety and depression, whereas the most intensified stress levels were observed in a population from Europe [24].

The researchers from India conducted a study on a date after the lockdown that was similar to ours since it was four and six weeks, respectively [53]. Surveys were distributed in both countries during the end of the lockdown period. The results showed that students from both countries differed significantly in terms of comparing the number of students experiencing the severity of emotional disorders from moderate to extremely severe. In the population of Polish students, depression and anxiety were mostly enhanced, while anxiety dominates in the profile of emotional distress in Indian students. Perhaps these differences can be explained by the large disparity in the rates of the number of infected and the number of deaths per million.

Researchers from Bangladesh achieved a surprising result, especially considering that Bangladesh had the lowest mortality and infection rates among all compared countries. Namely, Bangladeshi students reported the highest percentage level in the subscale from moderate to extremely severe stress [54]. Furthermore, students from Bangladesh showed significantly higher levels of depression, stress, and anxiety compared to Poland and Spain. DASS-21 scores were significantly higher among women aged 25 to 29 who live in urban areas, who were dissatisfied with their sleep, spent more hours browsing the Internet, were dissatisfied with academic studies in the current COVID-19 circumstances, and smoked. In each of the compared countries, women completed the survey more often than men. Only in the Bangladeshi population, the majority of respondents were men; nevertheless, female students showed higher levels of emotional distress similarly to all compared countries [53]. These results suggest that women, despite the country of origin, are more vulnerable to experience enhanced depressive, anxiety, and stress symptoms.

From all the above-analyzed countries, an unexpected situation was observed in Spain, where the highest rates of total confirmed cases and deaths of COVID-19 per 1 million population were noted. The percentage of students showing clinically significant levels of depression and stress was similar in the Spanish and Indian studies, although these countries significantly differed in terms of the mortality rates and the number of infections [38,53]. Comparing to Polish students, the respondents from Spain showed significantly lower results in all of the DASS-21 subscales (depression, anxiety, and stress). This observation indicates that there might be many different variables that may affect mental health except for the ones associated with COVID-19.

In our study, females showed statistically higher emotional distress levels compared to males. Generally, depressive [55] and anxiety [56,57] symptoms are more prevalently observed in females; therefore, increased levels of emotional distress in females compared to males seem not to be surprising.

Regarding the field of study, the greatest emotional response was shown by students of arts and humanities in both Poland and Spain; Spanish students who study either arts and humanities or social sciences and law presented the highest depression, anxiety, and stress levels [38]. In our study, we observed that being a science (OR = 2.04) or an art and humanities student (OR = 1.98) was associated with approximately 2-fold higher odds of more intensified total emotional distress. It is also worth emphasizing that the lowest intensity of the emotional response in both studies was observed among medical students, which is a very favorable phenomenon in the context of the nature of their future professional work and the potential risk of exposure to various stressors, including those related to the pandemic.

The results of our study showed that ‘living with roommates’ (OR = 1.25) constituted one of the risk factors that considerably intensifies the emotional distress among students. Such a high frequency of this chosen answer could be associated with numerous factors such as different emotional reactions of the roommates and their behaviors that could possibly cause negative thoughts and feelings in the respondents. Besides, it is worth noting that the students who were living with roommates could additionally worry about their family members or friends who were not living with them during the pandemic, contributing to the increased emotional distress of this group. Cao et al. pointed out that living with parents could potentially constitute a protective factor against anxiety symptoms [17]. Additionally, not living with a family during a pandemic has been associated with a greater risk of reporting at least one mental health outcome [58]. Isolation from family and friends and living with roommates during the lockdown could increase the emotional distress of participants, which was also confirmed in the studies by Wathelet et al. and Wang et al. [58,59]. Nevertheless, we do not have additional information about who the roommates actually were and whether they were rather a support or an emotional burden for the respondents. However, when we take into consideration the fact that amongst nine of the most stress-related situations, ‘fear of infection of the loved ones’ was one of the most strongly correlated with the general intensity of anxiety, then the isolation from the family due to lockdown and living with a roommate (other than family or any close relatives) might be an additional factor intensifying emotional distress.

In our study, loneliness turned out to be the greatest difficulty for Polish students during the outbreak of the COVID-19 pandemic (OR = 293.31), which is generally considered as a risk factor implicated in either development or progression of depression [60]. Due to the introduction of epidemiological restrictions, loneliness might significantly contribute to the higher intensity of depressive symptoms. Moreover, there is evidence that the feeling of loneliness because of the COVID-19 pandemic is more experienced in young people [61,62]. Interestingly, Sundarasen et al. showed that loneliness contributed to the increase in the anxiety levels in the group of students from Malaysia [36]. In a meta-analysis, Loades et al. pointed out that there was an association between loneliness and/or social isolation and exacerbation of depressive symptoms, especially in childhood/adolescence; the researchers observed that intensified depressive symptoms are more pronounced in females rather than males [63].

In the studied group, the co-occurrence of any mental disorder was associated with higher levels of emotional distress, depression, anxiety, as well as stress. However, we cannot completely assume whether such high levels of the above-mentioned variables were due to the pandemic itself or whether they were increased at baseline (before the outbreak of the pandemic); it was shown that generally, high levels of stress were related to numerous mental disorders, at the same time increasing the intensity of depressive and anxiety symptoms [64,65]. Those who used psychological and/or psychiatric support before the outbreak of the pandemic also showed significantly higher levels of emotional distress along with all of the DASS-21 subscales. Our results are consistent with those obtained by Vindegaard et al., who indicated that people who had preexisting psychiatric disorders are reported to experience worsening psychiatric symptoms during the COVID-19 pandemic [66]. Like the above-mentioned information, it is speculative whether it was associated with the pandemic or due to potentially increased depression and/or anxiety and/or stress symptoms at baseline of the possible psychological or psychiatric condition. Moreover, the observed relationship should be interpreted with caution; anxiety and depressive symptoms could be potentially intensified by the fear of illness and increased loneliness during the pandemic, respectively. It is extremely important for health workers to be aware of such associations, especially during the pandemic.

Regarding the economic situation, the respondents with stable family income presented the lowest emotional distress levels, contrary to those who had to start borrowing money during the pandemic. Low income is generally associated with greater psychological distress; therefore, the results of our study seem consistent [67]. Although the majority of the respondents were not working (neither physically nor mentally), the remaining (those who were working during the pandemic) mostly presented with increased depression levels. Working mentally or physically during the pandemic was related to a 0.5 and 0.4-fold lower odds of increased overall emotional distress, respectively. Therefore, it might be assumed that having a job by the students could be a potentially protective factor against increased overall emotional distress.

Already during the pandemic, Larionov and Mudło-Głagolska (2020) conducted a study on the Polish population and showed that females, families with a household of at least two persons, persons with children, unemployed individuals, and those with chronic diseases were at risk of a stronger emotional response during a pandemic [68]. The researchers presented percentage results that were quite close to those obtained in our study, although the average age of their respondents was 35.15 years (SD = 12.53). The DASS total score for this group was equal to 35.89 ± 33.74. In the depression subscale, the percentage of respondents ranging from moderate to extreme severe was 37.25%, whereas, in our study, it was 43.40%. On the anxiety subscale, 39.08% of respondents presented with moderate to extremely severe anxiety, and in our study, it was 27.3% of students. In the above-mentioned study, 34.12% of respondents ranged from moderate to extremely severe on the stress subscale, compared to 41% of the students in our study. The conclusion is that in the group with higher age, the intensity of anxiety was more intense than in the group of students.

Islam et al. (2020) showed that the male gender, living in the countryside, having satisfactory sleep (7–8 h per day), low Internet use (less than 2 h a day), and physical exercise might constitute potential protective factors against emotional distress. The authors also showed that tobacco smoking might be associated with higher levels of depression, anxiety, and/or stress and thus might constitute one of the potential risk factors. Besides, living in a nuclear family was assumed to be a potential risk factor for depression and stress [54]. It should be taken into consideration that the results of all of these studies differ due to several reasons. Firstly, it was because all of the surveys were launched on different dates and the time that had passed from the start of the lockdown in a particular country also differed, and the release of the survey was not standardized. Thus, the impact of SARS-CoV-2 spread on the mental health of the respondents might differ. What is more, the results might differ because of the restrictions introduced by a particular country that might be more or less strict and severe depending on both—the decisions of the government as well as the time when the survey was performed since the expansion of particular restrictions also differ in time. What could also affect the respondents’ reactions and depression, anxiety, and stress levels could be the form of providing information for the societies that might differ between the local social media.

## 9. Limitations of the Study

One of the major limitations of this study is that the respondents involved in this study (*n* = 2172) were only Polish students; therefore, these results cannot be generalized to other nationalities, racial, or ethnic groups. Among them, the majority of the respondents were females (*n* = 1585) and medical students (*n* = 1314). Therefore, it is hard to establish the obtained results as generalizable because the group of students involved in this study was slightly limited.

The reliability of the results was also limited by the fact that the study was conducted in the form of an online survey where the researchers could not assess the reliability of the information provided by the respondents. Moreover, the other limitation is that the students who fulfilled our survey were only those who were interested in the potential consequences of the COVID-19 pandemic on mental health, had access to the Internet, and were simply interested in taking part in such an online questionnaire.

Besides, it should be taken into consideration that the comparison of our results with other countries (Spain, India, China, and Bangladesh) based on the DASS-21 scale should be analyzed with caution since there might be many potential reasons for any differences between those countries including cultural bias of reporting on mental health.

Another limitation is the fact that the results of the obtained study reflect the well-being of students only during the few days when the survey was conducted without any insight into longitudinal effects on students’ mental health. On the other hand, the mental health status and psychological symptoms of the respondents before the COVID-19 pandemic were unknown to the researchers, which was associated with the potential difficulties in the interpretation of the obtained results as well.

Taking all the above facts into consideration, it must be stated that the obtained results cannot be assigned to the entire population of Polish students due to the profile of the student who most often completed the survey, the form of the survey which was carried out as an online questionnaire, and the time in which it was conducted that only reflect the well-being of Polish students in the short and particular period of the pandemic. It would be beneficial to perform further studies, especially longitudinal ones, which could provide a clearer understanding of the pandemic effect on students’ depression, anxiety, and stress levels.

## 10. Conclusions

Our study was conducted six weeks after the lockdown in Poland when the restrictions were highly pronounced, the prevalence and the mortality rates were relatively low, and the knowledge about COVID-19 was still insufficient. The results of this study show that the severity of symptoms that range from moderate to extremely severe concerned the following groups of students—43.4% in the depression subscale, 27.3% in the anxiety subscale, and 41.0% in the stress subscale, indicating a high percentage of the students experienced significant clinical, emotional distress.

We tried to create a portrait of a Polish student who may potentially require specific psychological and/or psychiatric support during a pandemic. The results obtained in our study show that being a female science student, living with roommates, suffering from mental disorders, and using the support of a psychiatrist and/or psychologist before the pandemic predisposes a student to increased emotional response. Besides, additional aspects associated with the increased risk of enhanced emotional responses were a feeling of loneliness during the pandemic and a deterioration of the financial situation during the pandemic that required the need to borrow money to support oneself.

While comparing our results with other similar studies conducted during the lockdown with the usage of the same instrument—the DASS-21—it turned out that Polish students presented with higher depression, anxiety, and stress levels compared to Chinese and Spanish students, whereas they presented with lower levels compared to Bangladeshi students, indicating that socio-political factors might also potentially increase the emotional distress, and should be considered. Therefore, it must be taken into consideration that a particular nationality or culture might present different intensities of depression, anxiety, stress, or other emotional symptoms in response to the same agent. The understanding of this point is crucial to provide the right approach as well as proper potential psychological and/or psychiatric help for all of the students of different nationalities.

The social, economic, and health situations that are continually changing due to the ongoing COVID-19 pandemic in Poland and the world that require specific adaptive resources and social support might be a huge stressor to maintain the proper mental well-being of many students. Therefore, it is crucial to monitor the mental health status of students as well as to identify potential risk factors that might contribute to the induction of mental disorders to further provide the proper psychological and psychiatric help for those students who require it the most.

## Figures and Tables

**Table 1:** The severity labels used to describe the range of scores in the population of the DASS-21 multiplied by 2.

	Depression	Anxiety	Stress
Normal	0–8	0–7	0–14
Mild	8–13	8–9	15–18
Moderate	14–20	10–14	19–25
Severe	21–27	15–19	26–33
Extremely severe	28+	20+	34+

**Table 2:** Sociodemographic characteristics of the respondents included in the study.

Question	Answer	Number of Respondents	% of Respondents
Sex	Women	1585	73.0
	Men	587	27.0
Field of study	Arts and humanities	110	5.0
	Sciences	96	4.4
	Medicine	1314	60.5
	Engineering	219	10.0
	Social sciences	416	19.2
Year of study	I	511	23.5
	II	444	20.4
	III	507	23.3
	IV	322	14.8
	V	277	12.8
	VI	106	4.9
Place of residence (number of inhabitants) [in thousands]	Village	497	22.9
	Less than 20	219	10.1
	20–100	344	15.8
	100–300	276	12.7
	300–600	409	18.8
	More than 600	427	19.7
Marital status	Single	1426	65.6
	Informal relationship	667	30.7
	Married	54	2.5
Do you have children?	No	2130	98.1
	One child	20	0.9
I live with:	Alone	231	10.6
	Parents	1049	48.3
	Roommates	565	26.0
	Partner or spouse	301	13.9
	Partner/spouse and children	21	1.0

**Table 3:** Factors associated with the health status of respondents.

Question	Answer	Number of Respondents	% of Respondents
Did you get COVID-19?	No	2112	97.2
	Yes	28	1.3
Did any of your relatives/friends get COVID-19?	No	1943	89.5
	Yes, a family member	52	2.4
	Yes, a friend	161	7.4
Did any of your relatives/friends die because of COVID-19?	No	2142	98.6
	Yes, a family member	12	0.6
	Yes, a friend	14	0.6
Are you actively joining the fight against the COVID-19 epidemic?	No	1733	79.8
	Yes	439	20.2
Did you use psychological/psychiatric help before the beginning of the pandemic?	No	1800	82.9
	Yes, I used psychological support	162	7.5
	Yes, I used psychiatric support	80	3.7
	Yes, I used psychological and psychiatric support	130	6.0
Do you take any supplements/medicines that increase immunity?	No	1506	69.3
	Yes	665	30.6
What was most difficult for you during the pandemic?	Changes awaiting the world after the pandemic	440	20.3
	Change of the lifestyle	265	12.2
	Fear of being infected	66	3.0
	Fear of infection of the loved ones	728	33.5
	Financial instability	149	6.9
	Isolation	211	9.7
	Loneliness	149	6.9
Do you have any chronic disease?	No	1837	84.6
	Allergy	32	1.5
	Asthma	54	2.5
	Diabetes	15	0.7
	Mental disorders	41	1.9
	Thyroid diseases	88	4.1
	Other	105	4.8

**Table 4:** The economic situation of the respondents.

Question	Answer	Number of Respondents	% of Respondents
Did you work before the pandemic outbreak?	I did not work	1469	67.6
	I worked mentally	356	16.4
	I worked physically	316	14.5
	I ran my own business	30	1.2
Do you work currently?	No, I do not work	1799	82.8
	I work mentally	249	11.5
	I work physically	96	4.4
	I run my own business	28	1.3
How do you assess your economic situation during the pandemic?	I have a stable family income, nothing has changed	1278	58.8
	I have a stable family income, but the situation is worse than before	647	29.8
	I have to start using savings	191	8.8
	I have to borrow money from my family/friends during the outbreak of the pandemic because I do not have enough money to support myself	21	1.0
	I barely have enough money for living	27	1.2

**Table 5:** The number of students presenting a severity ranges of overall emotional distress, depression, anxiety, and stress levels.

	Normal		Mild		Moderate		Severe		Extremely Severe	
	*n*	%	*n*	%	*n*	%	*n*	%	*n*	%
**Depression**	948	43.6	282	13	432	19.9	221	10.2	289	13.3
**Anxiety**	1307	60.2	273	12.5	195	9.0	144	6.6	253	11.7
**Stress**	1026	47.2	255	11.8	333	15.3	364	16.8	194	8.9

**Table 6:** The comparison of the results obtained applying the DASS-21 scale on a population of students in the studies performed in Poland, Spain, China, India, and Bangladesh.

Country		Poland		Spain	China	India	Bangladesh
Date of the start and closure of the survey		20 April–26 April		28 March–4 April	31 January–2 February	23 April–30 April	11 April–24 April
The time that has from the confinement till the start of the survey		6 weeks		2 weeks	3 days after the WHO announced COVID-19 as a public health emergency	One month	2 weeks
Number of the respondents		2172		3707	1210	500	3122
Females (%)		73		66.1	67.3	65	40.5
Males (%)		27		33.9	32.7	35	59.5
Mean age/age range		22.1 ± 2.2		27.9 ± 12.4	12–21.4—28.4% 21.4–30.8—53.2% 30.8–40.2—7.8% 40.2–49.6—7.4% 49.6–59—3.2%	21.2 ± 1.3	21.4 ± 2
Mean and SD of the DASS-21 total score **		38.13 ± 26.51		ND	20.16 ± 20.42	ND	ND
DASS-21 (mean and SD for each of the subscales) ***	Depression	14.04 ± 10.44		5.52 ± 4.92	ND	ND	17.4 ± 10.7
	Anxiety	7.71 ± 8.29		3.34 ± 3.87*	ND	ND	13.8 ± 9.8
	Stress	16.93 ± 10.98		6.81 ± 4.72	ND	ND	21.3 ± 11
DASS-21 (% of the respondents with a particular degree of severity of depression, anxiety, and stress)	Depression	Normal	43.6	ND	69.7	57.5	23.9
		Mild	13.0		13.8	8.5	13.2
		Moderate	19.9		12.2	8.0	27.7
		Severe	10.2		4.3 *	8.0	15.5
		Extremely severe	13.3			18.0	19.7
	Anxiety	Normal	60.2		63.6	53.0	28.5
		Mild	12.5		7.5	5.0	7.9
		Moderate	9.0		20.4	10.5	23.3
		Severe	6.6		8.4 *	4.0	12.8
		Extremely severe	11.7			27.5	27.5
	Stress	Normal	47.2		67.9	68.0	29.9
		Mild	11.8		24.1	4.0	11.5
		Moderate	15.3		5.5	9.0	20.9
		Severe	16.8		2.6 *	6.5	21.2
		Extremely severe	8.9			12.5	16.5

ND—no data. * collectively for severe and extremely severe. ** the results of the analysis of the significance of the differences for DASS total score—Poland vs. China: *t* = 20,441, *p* < 0.0001, 95% CI: 19.69 to 16.246, Cohen’s *d* = 0.759 (medium). *** the results of the analysis of the significance of the differences for DASS Depression—Poland vs. Spain: *t* = 42,313, *p* < 0.0001, 95% CI: 8.914–8.125, Cohen’s *d* = 1.044 (large)—Poland vs. Bangladesh: *t* = −11.351, *p* < 0.0001, 95% CI: 2.779–3.94, Cohen’s *d* = 1.868 (large) Spain vs. Bangladesh: *t* = −60,438, *p* < 0.0001, 95% CI: 11.494–12.265, Cohen’s *d* = 1501 (large). *** the results of the analysis of the significance of the differences for DASS Anxiety—Poland vs. Spain: *t* = 27.402, *p* < 0.0001, 95% CI: 4.682–4.057, Cohen’s *d* = 0.675 (medium)—Poland vs. Bangladesh: *t* = −23.664, *p* < 0.0001, 95% CI: 5.585–6.594, Cohen’s *d* = 1.072 (large)—Spain vs. Bangladesh: *t* = −59.694, *p* < 0.0001, 95% CI: 10.116–10.803, Cohen’s *d* = 0.694, (medium). *** the results of the analysis of the significance of the differences for DASS Stress—Poland vs. Spain: *t* = 10.120, *p* < 0.0001, 95% CI: 10.525–9.745, Cohen’s *d* = 1.197 (large)—Poland vs. Bangladesh: *t* = −14.229, *p* < 0.0001, 95% CI: 3.767–4.972, Cohen’s *d* = 1.906 (large)—Spain vs. Bangladesh: *t* = −72.654, *p* < 0.0001, 95% CI: 14.099–14.881, Cohen’s *d* = 1.401 (large).

## Supplementary Materials

The following are available online at https://www.mdpi.com/2077-0383/10/5/944/s1, Table S1: The results of the DASS total score for the field of study, Table S1.2: The results of the DASS depression scale for the field of study, Table S1.3: The results of the DASS anxiety scale for the field of study, Table S1.4: The results of the DASS stress scale for the field of study, Table S2: The results of the DASS total score for the most prevalent difficulties during pandemics, Table S2.2: The results of the DASS depression scale for the most prevalent difficulties during pandemics, Table S2.3: The results of the DASS anxiety scale for the most prevalent difficulties during pandemics, Table S2.4: The results of the DASS stress scale for the most prevalent difficulties during pandemics, Table S3: The results of the DASS total score for the most prevalent chronic diseases, Table S3.2: The results of the DASS depression scale for the most prevalent chronic diseases, Table S3.3: The results of the DASS anxiety scale for the most prevalent chronic diseases, Table S3.4: The results of the DASS stress scale for the most prevalent chronic diseases, Table S4: The results of the DASS total score for the usage of either psychological or psychiatric help before the pandemics outbreak, Table S4.2: The results of the DASS depression scale for the usage of either psychological or psychiatric help before the pandemics outbreak, Table S4.3: The results of the DASS anxiety scale for the usage of either psychological or psychiatric help before the pandemics outbreak, Table S4.4: The results of the DASS stress scale for the usage of either psychological or psychiatric help before the pandemics outbreak, Table S5: The results of the DASS total score for the economic situation during pandemics, Table S5.2: The results of the DASS depression scale for the economic situation during pandemics, Table S5.3: The results of the DASS anxiety scale for the economic situation during pandemics, Table S5.4: The results of the DASS stress scale for the economic situation during pandemics, Table S6: The results of the DASS total score for the current employment situation, Table S6.2: The results of the DASS depression scale for the current employment situation, Table S6.3: The results of the DASS anxiety scale for the current employment situation, Table S6.4: The results of the DASS stress scale for the current employment situation, Table S7: The results of the DASS total score for the current living situation, Table S7.2: The results of the DASS depression scale for the current living situation, Table S7.3: The results of the DASS anxiety scale for the current living situation, Table S7.4: The results of the DASS stress scale for the current living situation, Table S8: The sociodemographic data of the respondents.

## Abbreviations

COVID-19	Coronavirus disease 2019
DASS-21	Depression, Anxiety, and Stress Scale-21 Items
M	Mean
Me	Median
MERS-CoV	Middle East Respiratory Syndrome Coronavirus
OR	Odds ratio
SARS-CoV	Severe Acute Respiratory Syndrome Coronavirus
SARS-CoV-2	Severe Acute Respiratory Syndrome Coronavirus-2
WHO	World Health Organization

## References

[1] Lu H., Stratton C.W., Tang Y.W. (2020). Outbreak of pneumonia of unknown etiology in Wuhan, China: The mystery and the miracle. J. Med. Virol..

[2] Huang C., Wang Y., Li X., Ren L., Zhao J., Hu Y., Zhang L., Fan G., Xu J., Gu X. (2020). Clinical features of patients infected with 2019 novel coronavirus in Wuhan, China. Lancet.

[3] World Health Organization Rolling Updates On Coronavirus Disease (COVID-19) [Internet]. https://www.who.int/emergencies/diseases/novel-coronavirus-2019/events-as-they-happen.

[4] World Health Organization WHO Director-General’s Opening Remarks at the Media Briefing on COVID-19–11 March 2020 [Internet]. https://www.who.int/dg/speeches/detail/who-director-general-s-opening-remarks-at-the-media-briefing-on-covid-19---11-march-2020.

[5] Di Gennaro F., Pizzol D., Marotta C., Antunes M., Racalbuto V., Veronese N., Smith L. (2020). Coronavirus Diseases (COVID-19) Current Status and Future Perspectives: A Narrative Review. Int. J. Environ. Res. Public Health..

[6] Nadeem M.S., Zamzami M.A., Choudhry H., Murtaza B.N., Kazmi I., Ahmad H., Shakoori A.-R. (2020). Origin, Potential Therapeutic Targets and Treatment for Coronavirus Disease (COVID-19). Pathogens.

[7] Mungroo M.R., Khan N.A., Siddiqui R. (2020). Novel Coronavirus: Current Understanding of Clinical Features, Diagnosis, Pathogenesis, and Treatment Options. Pathogens.

[8] Baj J., Karakuła-Juchnowicz H., Teresiński G., Buszewicz G., Ciesielka M., Sitarz E., Forma A., Karakuła K., Flieger W., Portincasa P. (2020). COVID-19: Specific and Non-Specific Clinical Manifestations and Symptoms: The Current State of Knowledge. J. Clin. Med..

[9] Grant B.W., Lahore H., McDonnell L.S., Baggerly A.C., French B.C., Aliano L.J., Bhattoa H.P. (2020). Evidence that Vitamin D Supplementation Could Reduce Risk of Influenza and COVID-19 Infections and Deaths. Nutrients.

[10] World Health Organization Coronavirus Disease (COVID-19) Pandemic [Internet]. https://www.who.int/emergencies/diseases/novel-coronavirus-2019.

[11] British Broadcasting Company Coronavirus: The World in Lockdown in Maps and Charts [Internet]. https://www.bbc.com/news/world-52103747.

[12] Government of Poland Mapa Zarażeń Koronawirusem (SARS-CoV-2). [Map of coronavirus infection (SARS-CoV-2).] [Internet]. https://www.gov.pl/web/koronawirus/wykaz-zarazen-koronawirusem-sars-cov-2.

[13] Government of Poland Wprowadzamy Stan Epidemii w Polsce [Introducing the Epidemic State in Poland] [Internet]. https://www.gov.pl/web/koronawirus/wprowadzamy-stan-epidemii-w-polsce.

[14] Government of Poland Kolejne Kroki W Walce Z Koronawirusem–W Sklepie Mniej Osób, Ograniczenia W Poruszaniu Nieletnich, a Parki, Plaże I Bulwary Zamknięte. [Next Steps in the Fight Against Coronavirus-fewer People in the Store, Restrictions on the Movement of Minors, and Parks, Beaches and Boulevards closed.] [Internet]. https://www.gov.pl/web/koronawirus/kolejne-kroki.

[15] Wang C., Pan R., Wan X., Tan Y., Xu L., Ho S.C., Ho R.C. (2020). Immediate Psychological Responses and Associated Factors during the Initial Stage of the 2019 Coronavirus Disease (COVID-19) Epidemic among the General Population in China. Int. J. Environ. Res. Public Health.

[16] Lee M., You M. (2020). Psychological and Behavioral Responses in South Korea During the Early Stages of Coronavirus Disease 2019 (COVID-19). Int. J. Environ. Res. Public Health.

[17] Cao W., Fang Z., Guoqiang H., Han M., Xu X., Dong J., Zheng J. (2020). The psychological impact of the COVID-19 epidemic on college students in China. Psychiatry Res..

[18] He J., He L., Zhou W., Nie X., He M. (2020). Discrimination and Social Exclusion in the Outbreak of COVID-19. Int. J. Environ. Res. Public Health.

[19] Sim K., Chan Y., Phui-Nah C., Chua H., Soon S. (2010). Psychosocial and coping responses within the community health care setting towards a national outbreak of an infectious disease. J. Psychosom. Res..

[20] Saeed S.A., Hebishi K. (2020). The psychiatric consequences of COVID-19: 8 Studies. Curr. Psychiatry.

[21] Pérez-Fuentes D.M., Molero Jurado D.M., Oropesa Ruiz F.N., Martos Martínez Á., Simón Márquez D.M., Herrera-Peco I., Linares J.J.G. (2020). Questionnaire on Perception of Threat from COVID-19. J. Clin. Med..

[22] Nguyen C.H., Nguyen H.M., Do N.B., Tran Q.C., Nguyen T.P.T., Pham M.K., Pham L.V., Tran K.V., Duong T.T., Tran T.V. (2020). People with Suspected COVID-19 Symptoms Were More Likely Depressed and Had Lower Health-Related Quality of Life: The Potential Benefit of Health Literacy. J. Clin. Med..

[23] Huang Y., Zhao N. (2020). Generalized anxiety disorder, depressive symptoms and sleep quality during COVID-19 epidemic in China: A web-based cross-sectional survey. Psychiatry Res..

[24] Salari N., Hosseinian-Far A., Jalali R., Vaisi-Raygani A., Rasoulpoor S., Mohammadi M., Rasoulpoor S., Khaledi-Paveh B. (2020). Prevalence of stress, anxiety, depres- sion among the general population dur- ing the COVID-19 pandemic: A systematic review and meta-analysis. Glob. Health..

[25] Xiong J., Lipsitz O., Nasri F., Lui L.M.W., Gill H., Phan L., Chen-Li D., Iacobucci M., Ho R., Majeed A. (2020). Impact of COVID-19 pandemic on mental health in the general population: A systematic review. J. Affect. Disord..

[26] Lakhan R., Agrawal A., Sharma M. (2020). Prevalence of Depression, Anxiety, and Stress during COVID-19 Pandemic. J. Neurosci. Rural. Pr..

[27] Pieh C., Budimir S., Probst T. (2020). The effect of age, gender, income, work, and physical activity on mental health during coronavirus disease (COVID-19) lockdown in Austria. J. Psychosom. Res..

[28] García-Fernández L., Romero-Ferreiro V., López-Roldán P.D., Padilla S., Rodriguez-Jimenez R. (2020). Mental Health in Elderly Spanish People in Times of COVID-19 Outbreak. Am. J. Geriatr. Psychiatry.

[29] Mamun M.A., Akter S., Hossain I., Faisal M.T.H., Rahman A., Arefin A., Khan I., Hossain L., Haque A., Hossain S. (2020). Financial threat, hardship and distress predict depression, anxiety and stress among the unemployed youths: A Bangladeshi multi-city study. J. Affect. Disord..

[30] Achdut N., Refaeli T. (2020). Unemployment and Psychological Distress among Young People during the COVID-19 Pandemic: Psychological Resources and Risk Factors. Int. J. Environ. Res. Public Health.

[31] Wang C., Chudzicka-Czupała A., Grabowski D., Pan R., Adamus K., Wan X., Hetnał M., Tan Y., Olszewska-Guizzo A., Xu L. (2020). The Association Between Physical and Mental Health and Face Mask Use During the COVID-19 Pandemic: A Comparison of Two Countries With Different Views and Practices. Front. Psychiatry.

[32] Kazmi S.S.H., Hasan K., Talib S., Saxena S. (2020). COVID-19 and Lockdwon: A Study on the Impact on Mental Health. https://ssrn.com/abstract=3577515.

[33] Wu M., Xu W., Yao Y., Zhang L., Guo L., Fan J., Chen J. (2020). Mental health status of students’ parents during COVID-19 pandemic and its influence factors. Gen Psychiatr..

[34] Epifanio M.S., Andrei F., Mancini G., Agostini F., Piombo M.A., Spicuzza V., Riolo M., Lavanco G., Trombini E., La Grutta S. (2021). The Impact of COVID-19 Pandemic and Lockdown Measures on Quality of Life among Italian General Population. J. Clin. Med..

[35] Hoyt L.T., Cohen A.K., Dull B., Castro E.M., Yazdani N. (2020). “Constant Stress Has Become the New Normal”: Stress and Anxiety Inequalities Among, U.S. College Students in the Time of COVID-19. J. Adolesc. Health.

[36] Sundarasen S., Chinna K., Kamaludin K., Nurunnabi M., Baloch G.M., Khoshaim H.B., Hossain S.F.A., Sukayt A. (2020). Psychological Impact of COVID-19 and Lockdown among University Students in Malaysia: Implications and Policy Recommendations. Int. J. Environ. Res. Public Health..

[37] Li Y., Wang Y., Jiang J., Valdimarsdóttir U.A., Fall K., Fang F., Song H., Lu D., Zhang W. (2020). Psychological distress among health professional students during the COVID-19 outbreak. Psychol. Med..

[38] Dratva J., Zysset A., Schlatter N., von Wyl A., Huber M., Volken T. (2020). Swiss University Students’ Risk Perception and General Anxiety during the COVID-19 Pandemic. Int. J. Environ. Res. Public Health..

[39] Odriozola-González P., Planchuelo-Gómez Á., Irurtia M.J., de Luis-García R. (2020). Psychological effects of the COVID-19 outbreak and lockdown among students and workers of a Spanish university. Psychiatry Res..

[40] Polish Translation of DASS. http://www2.psy.unsw.edu.au/Groups/Dass/Polish/Polish.htm.

[41] DASS Translations. http://www2.psy.unsw.edu.au/groups/dass/translations.htm.

[42] Lovibond S.H., Lovibond P.F. (1995). Manual for the Depression Anxiety Stress Scales.

[43] Antony M.M., Bieling P.J., Cox B.J., Enns M.W., Swinson R.P. (1998). Psychometric properties of the 42-item and 21-item versions of the depression anxiety stress scales in clinical groups and a community sample. Psychol. Assess..

[44] Depression Anxiety Stress Scales–Short Form (DASS-21) [Internet]. https://novopsych.com.au/assessments/depression-anxiety-stress-scales-short-form-dass-21/.

[45] Ruiz F., Garcia-Martin M., Suárez F., Juan C., Odriozola-González P. (2017). The hierarchical factor structure of the Spanish version of the Depression Anxiety and Stress Scale-21 (DASS-21). Int. J. Psychol. Psychol. Ther..

[46] Henry J.D., Crawford J.R. (2005). The short-form version of the Depression Anxiety Stress Scales (DASS-21): Construct validity and normative data in a large non-clinical sample. Br. J. Clin. Psychol..

[47] Steel Z., Marnane C., Iranpour C., Chey T., Jackson J.W., Patel V., Silove D. (2014). The global prevalence of common mental disorders: A systematic review and meta-analysis 1980–2013. Int. J. Epidemiol..

[48] Shah S.M.A., Mohammad D., Quereshi M.F.H., Abbas M.Z., Aleem S. (2021). Prevalence, Psychological Responses and Associated Correlates of Depression, Anxiety and Stress in a Global Population, During the Coronavirus Disease (COVID-19) Pandemic. Community Ment. Health J..

[49] Zawislak D., Zur-Wyrozumska K., Habera M., Skrzypiec K., Pac A., Cebula G. (2020). Evaluation of a Polish Version of the Depression Anxiety Stress Scales (DASS-21). J. Neurosci. Cogn. Stud..

[50] Martinotti G., Alessi M.C., Di Natale C., Sociali A., Ceci F., Lucidi L., Picutti E., Di Carlo F., Corbo M., Vellante F. (2020). Psychopathological Burden and Quality of Life in Substance Users During the COVID-19 Lockdown Period in Italy. Front. Psychiatry.

[51] Moroń M., Biolik-Moroń M. (2021). Trait emotional intelligence and emotional experiences during the COVID-19 pandemic outbreak in Poland: A daily diary study. Personal. Individ. Differ..

[52] Selye I. (1946). The general adaptation syndrome and the diseases of adaptation. J. Allergy..

[53] Suryadevara V., Adusumalli C., Adusumilli P.K., Chalasani S.H., Radhakrishnan R. (2020). Mental Health Status among the South Indian Pharmacy Students during Covid-19 Pandemic Quarantine Period: A Cross-Sectional Study. https://www.x-mol.com/paper/1260295362728624128.

[54] Islam M., Sujan S.H., Tasnim R., Sikder T., Potenza M.N., Os J.V. (2020). Psychological Responses during the COVID-19 Outbreak among University Students in Bangladesh. https://psyarxiv.com/cndz7/.

[55] Lim G.Y., Tam W.W., Lu Y., Ho C.S., Zhang M.W., Ho R.C. (2018). Prevalence of Depression in the Community from 30 Countries between 1994 and 2014. Sci. Rep..

[56] McLean C.P., Asnaani A., Litz B.T., Hofmann S.G. (2011). Gender differences in anxiety disorders: Prevalence, course of illness, comorbidity and burden of illness. J. Psychiatr. Res..

[57] Bahrami F., Yousefi N. (2011). Females are more anxious than males: A metacognitive perspective. Iran. J. Psychiatry Behav. Sci..

[58] Wathelet M., Duhem S., Vaiva G., Baubet T., Habran E., Veerapa E., Debien C., Molenda S., Horn M., Grandgenèvre P. (2020). Factors Associated With Mental Health Disorders Among University Students in France Confined During the COVID-19 Pandemic. JAMA Netw. Open.

[59] Wang X., Hegde S., Son C., Keller B., Smith A., Sasangohar F. (2020). Investigating Mental Health of US College Students During the COVID-19 Pandemic: Cross-Sectional Survey Study. J. Med. Internet. Res..

[60] Hawkley L.C., Cacioppo J.T. (2010). Loneliness matters: A theoretical and empirical review of consequences and mechanisms. Ann. Behav. Med..

[61] Losda-Baltar A., Jiménez-Gonzalo L., Gallego-Alberto L., Pedroso-Chaparro M.D.S., Fernandes-Pires J., Márquez-González M. (2021). “We’re Staying at Home”. Association of Self-Perceptions of Aging, Personal and Family Resources and Loneliness With Psychological Distress During the Lock-Down Period of COVID-19. J. Gerontol. B Psychol. Sci. Soc. Sci..

[62] Groarke J.M., Berry E., Graham-Wisener L., Mckenna-Plumley P.E., Mcglinchey E., Armour C. (2020). Loneliness in the UK during the COVID-19 pandemic: Cross-sectional results from the COVID-19 Psychological Wellbeing Study. PLoS ONE.

[63] Loades M.E., Chatburn E., Higson-Sweeney N., Reynolds S., Shafran R., Brigden A., Linney C., McManus M.N., Borwick C., Crawley E. (2020). Rapid Systematic Review: The Impact of Social Isolation and Loneliness on the Mental Health of Children and Adolescents in the Context of COVID-19. J. Am. Acad. Child Adolesc. Psychiatry.

[64] Herbert J. (1997). Stress, the brain, and mental illness. BMJ.

[65] Cattaneo A., Riva M.A. (2016). Stress-induced mechanisms in mental illness: A role for glucocorticoid signalling. J. Steroid. Biochem. Mol. Biol..

[66] Vindegaard N., Benros M.E. (2020). COVID-19 pan-demic and mental health consequences: Sys-tematic review of the current evidence. Brain. Behav. Immun..

[67] Orpana H.M., Lemyre L., Gravel R. (2009). Income and psychological distress: The role of the social environment. Health Rep..

[68] Larionov P., Mudło-Głagolska K. (2020). Mental Health Risk Factors during COVID-19 Pandemic in the Polish Population. https://psyarxiv.com/3ku8w/.

