# Supplementary material for: The Outbreak of SARS-CoV-2 Pandemic and the Well-Being of Polish Students: The Risk Factors of the Emotional Distress during COVID-19 Lockdown
Source: J Clin Med. 2021 Feb 28;10(5):944. doi: 10.3390/jcm10050944 (PMC7957694; doi:10.3390/jcm10050944)
Supplement: Supplementary file 1 [file jcm-10-00944-s001.pdf]

Table S1: The results of the DASS total score for the field of study.

	Field of study							
	**Group**	**Mean**	**Median**	**SD**	**Kruskal–Wallis H test**	**Bonferroni correction**	**p-value**	**Multiple comparisons* (p-value)**
**DASS total score**	**I. Arts and humanities**	43.51	39.00	28.98	16.16	0.01	0.0028	II vs. III (0.009)
	**II. Sciences**	46.08	42.00	27.91				
	**III. Medicine**	36.56	31.00	25.49				
	**IV. Engineering**	38.24	32.00	26.79				
	**V. Social sciences**	39.65	35.00	28.14				

* only significant

Table S1.2: The results of the DASS depression scale for the field of study.

	Field of study							
	**Group**	**Mean**	**Median**	**SD**	**Kruskal–Wallis H test**	**Bonferroni correction**	**p-value**	**Multiple comparisons* (p-value)**
**DASS Depression Scale**	**I. Arts and humanities**	15.42	13.00	10.92	19.82	0.01	0.0005	II vs. III (0.012)
	**II. Sciences**	16.65	16.00	11.44				
	**III. Medicine**	12.73	10.00	10.01				
	**IV. Engineering**	14.74	12.00	11.15				
	**V. Social sciences**	13.93	12.00	10.80				

* only significant

Table S1.3: The results of the DASS anxiety scale for the field of study.

	Field of study							
	**Group**	**Mean**	**Median**	**SD**	**Kruskal–Wallis H test**	**Bonferroni correction**	**p-value**	**Multiple comparisons***
**DASS Anxiety Scale**	**I. Arts and humanities**	9.02	6.00	9.63	14.00	-	0.0073	-
	**II. Sciences**	9.67	6.00	9.21				
	**III. Medicine**	7.26	4.00	8.00				
	**IV. Engineering**	7.24	4.00	8.07				
	**V. Social sciences**	8.52	6.00	8.64				

* only significant

Table S1.4: The results of the DASS stress scale for the field of study.

Field of study								
**DASS Stress Scale**	**Group**	**Mean**	**Median**	**SD**	**Kruskal–Wallis H test**	**Bonferroni correction**	**p-value**	**Multiple comparisons***

	I. Arts and humanities	19.07	19.00	11.63	11.75	-	0.0193	-
	II. Sciences	19.77	20.00	11.12				
	III. Medicine	16.56	16.00	10.73				
	IV. Engineering	16.26	26.00	10.82				
	V. Social sciences	17.19	16.00	11.51				

* only significant

Table S2: The results of the DASS total score for the most prevalent difficulties during pandemics.

What was the most difficult for you during pandemics?								
	**Group**	**Mean**	**Median**	**SD**	**Kruskal–Wallis H test**	**Bonferroni correction**	**p-value**	**Multiple comparisons***
**DASS Total Score**	**I. Financial instability**	38.92	34.00	27.65	87.51	0.0055	0.0000	IV vs. VII (0.000)
	**II. Fear of infection of the loved ones**	36.05	32.00	24.42				
	**III. Isolation**	40.30	34.00	27.54				
	**IV. Changes awaiting the world after the pandemics**	35.69	30.00	25.47				III vs. IX (0.000)
	**V. Change of the lifestyle**	40.35	35.00	28.44				
	**VI. Concern about education**	35.02	28.00	27.46				
	**VII. Loneliness**	51.29	52.00	26.01				VII vs. IX (0.000)
	**VIII. Fear of being infected**	39.01	32.00	28.34				
	**IX. I was not afraid**	10.23	4.00	13.85				

* only significant

Table S2.2: The results of the DASS depression scale for the most prevalent difficulties during pandemics.

What was the most difficult for you during pandemics?								
	**Group**	**Mean**	**Median**	**SD**	**Kruskal–Wallis H test**	**Bonferroni correction**	**p-value**	**Multiple comparisons***
**DASS Depression Scale**	**I. Financial instability**	14.29	12.00	11.17	106.69	0.0055	0.000	IV vs. VII (0.000)
	**II. Fear of infection of the loved ones**	12.04	10.00	9.41				
	**III. Isolation**	14.30	12.00	10.25				

	**IV. Changes awaiting the world after the pandemics**	12.62	10.00	10.02				III vs. IX (0.000)
	**V. Change of the lifestyle**	14.81	12.00	11.27				
	**VI. Concern about education**	12.13	10.00	9.08				VII vs. IX (0.000)
	**VII. Loneliness**	12.38	20.00	11.3				
	**VIII. Fear of being infected**	12.32	10.00	9.74				
	**IX. I was not afraid**	4.85	2.00	7.09				

* only significant

Table S2.3: The results of the DASS anxiety scale for the most prevalent difficulties during pandemics.

**What was the most difficult for you during pandemics?**								
	**Group**	**Mean**	**Median**	**SD**	**Kruskal–Wallis H test**	**Bonferroni correction**	**p-value**	**Multiple comparisons***
**DASS Anxiety Scale**	**I. Financial instability**	7.82	6.00	8.31	53.84	0.0055	0.0000	III vs. IX (0.001)
	**II. Fear of infection of the loved ones**	7.58	6.00	7.67				
	**III. Isolation**	7.59	4.00	8.94				
	**IV. Changes awaiting the world after the pandemics**	7.13	4.00	7.90				VII vs. IX (0.000)
	**V. Change of the lifestyle**	7.67	4.00	8.88				
	**VI. Concern about education**	7.19	2.00	9.61				
	**VII. Loneliness**	9.94	8.00	8.38				VIII vs. IX (0.000)
	**VIII. Fear of being infected**	10.55	6.00	10.10				
	**IX. I was not afraid**	1.69	1.00	3.91				

* only significant

Table S2.4: The results of the DASS stress scale for the most prevalent difficulties during pandemics.

**What was the most difficult for you during pandemics?**								
**DASS Stress Scale**	**Group**	**Mean**	**Median**	**SD**	**Kruskal–Wallis H test**	**Bonferroni correction**	**p-value**	**Multiple comparisons***

	Group	Mean	Median	SD	Kruskal–Wallis H test	Bonferroni correction	p-value	Multiple comparisons*
	**I. Financial instability**	16.80	16.00	11.71	74.58	0.0055	0.0000	III vs. IX (0.000)
	**II. Fear of infection of the loved ones**	16.44	16.00	10.37				
	**III. Isolation**	18.41	18.00	11.34				
	**IV. Changes awaiting the world after the pandemics**	15.94	14.00	10.71				IV vs. IX (0.000)
	**V. Change of the lifestyle**	17.89	18.00	11.26				
	**VI. Concern about education**	15.70	14.00	11.51				
	**VII. Loneliness**	20.98	22.00	10.41				VII vs. IX (0.000)
	**VIII. Fear of being infected**	16.15	14.00	11.67				
	**IX. I was not afraid**	3.69	1.00	5.76				

* only significant

Table S3: The results of the DASS total score for the most prevalent chronic diseases.

**Do you have any chronic disease?**								
	**Group**	**Mean**	**Median**	**SD**	**Kruskal–Wallis H test**	**Bonferroni correction**	**p-value**	**Multiple comparisons***
**DASS total score**	**I. Mental disorders**	58.83	58.00	29.27	44.14	0.0071	0.0000	I vs. VII (0.000)
	**II. Thyroid diseases**	45.05	40.00	29.16				
	**III. Allergy**	43.63	37.00	24.81				
	**IV. Diabetes**	43.60	42.00	22.94				
	**V. Asthma**	44.82	41.00	28.09				
	**VI. Other**	43.96	40.00	25.48				
	**VII. No**	36.66	32.00	26.08				

* only significant

Table S3.2: The results of the DASS depression scale for the most prevalent chronic diseases.

**Do you have any chronic disease?**								
	**Group**	**Mean**	**Median**	**SD**	**Kruskal–Wallis H test**	**Bonferroni correction**	**p-value**	**Multiple comparisons***
**DASS Depression Scale**	**I. Mental disorders**	20.88	20.00	11.25	27.02	0.0071	0.008	I vs. VII (0.000)
	**II. Thyroid diseases**	14.96	12.00	11.56				
	**III. Allergy**	13.69	11.00	10.33				

	IV. Diabetes	15.47	14.00	10.60				
	V. Asthma	14.67	14.00	10.91				
	VI. Other	15.03	14.00	9.79				
	VII. No	13.12	10.00	10.32				

* only significant

Table S3.3: The results of the DASS anxiety scale for the most prevalent chronic diseases.

Do you have any chronic disease?								
	**Group**	**Mean**	**Median**	**SD**	**Kruskal–Wallis H test**	**Bonferroni correction**	**p-value**	**Multiple comparisons***
**DASS Anxiety Scale**	**I. Mental disorders**	14.05	12.00	11.68	43.30	0.0071	0.0000	I vs. VII (0.001)
	**II. Thyroid diseases**	10.23	8.00	9.68				
	**III. Allergy**	8.38	6.00	7.73				
	**IV. Diabetes**	7.73	6.00	7.17				
	**V. Asthma**	11.41	9.00	10.06				
	**VI. Other**	9.52	8.00	8.56				
	**VII. No**	7.22	4.00	7.96				

* only significant

Table S3.4: The results of the DASS stress scale for the most prevalent chronic diseases.

Do you have any chronic disease?								
	**Group**	**Mean**	**Median**	**SD**	**Kruskal–Wallis H test**	**Bonferroni correction**	**p-value**	**Multiple comparisons***
**DASS Stress Scale**	**I. Mental disorders**	23.90	24.00	11.68	42.42	0.0071	0.0000	I vs. VII (0.001)
	**II. Thyroid diseases**	19.86	20.00	11.37				
	**III. Allergy**	21.56	21.00	10.41				
	**IV. Diabetes**	20.40	22.00	9.14				
	**V. Asthma**	18.74	20.00	10.42				
	**VI. Other**	19.41	20.00	12.67				
	**VII. No**	17.15	8.00	10.33				

* only significant

Table S4: The results of the DASS total score for the usage of either psychological or psychiatric help before the pandemics outbreak.

Did you use psychological/psychiatric help before the beginning of the pandemics?								
**DASS Total Score**	**Group**	**Mean**	**Median**	**SD**	**Kruskal–Wallis H test**	**Bonferroni correction**	**p-value**	**Multiple comparisons***
	**I. No**	35.43	30.00	25.15	102.22	0.0125	0.000	I vs. II

	**II. Yes, I used psychological and psychiatric services**	56.71	56.00	29.53				(0.000)
								I vs. III (0.000)
	**III. Yes, I used psychological services**	46.51	43.00	28.12				
	**IV. Yes, I used psychiatric services**	51.60	49.00	28.55				I vs. IV (0.000)

* only significant

Table S4.2: The results of the DASS depression scale for the usage of either psychological or psychiatric help before the pandemics outbreak.

**Did you use psychological/psychiatric help before the beginning of the pandemics?**								
	**Group**	**Mean**	**Median**	**SD**	**Kruskal–Wallis H test**	**Bonferroni correction**	**p-value**	**Multiple comparisons***
**DASS Depression Scale**	**I. No**	12.56	10.00	9.94	78.21	0.0125	0.0000	I vs. II (0.000)
	**II. Yes, I used psychological and psychiatric services**	20.15	20.00	12.18				I vs. III (0.000)
	**III. Yes, I used psychological services**	15.83	14.00	10.55				I vs. IV (0.000)
	**IV. Yes, I used psychiatric services**	18.85	19.00	11.83				

* only significant

Table S4.3: The results of the DASS anxiety scale for the usage of either psychological or psychiatric help before the pandemics outbreak.

**Did you use psychological/psychiatric help before the beginning of the pandemics?**								
	**Group**	**Mean**	**Median**	**SD**	**Kruskal–Wallis H test**	**Bonferroni correction**	**p-value**	**Multiple comparisons***
**DASS Anxiety Scale**	**I. No**	6.87	4.00	7.66	92.20	0.0125	0.000	I vs. II (0.000)
	**II. Yes, I used psychological and psychiatric services**	13.26	12.00	10.43				I vs. III (0.000)
	**III. Yes, I used psychological services**	10.35	8.00	9.50				I vs. IV (0.000)
	**IV. Yes, I used psychiatric services**	12.05	10.00	9.63				

* only significant

Table S4.4: The results of the DASS stress scale for the usage of either psychological or psychiatric help before the pandemics outbreak.

Did you use psychological/psychiatric help before the beginning of the pandemics?								
	**Group**	**Mean**	**Median**	**SD**	**Kruskal–Wallis H test**	**Bonferroni correction**	**p-value**	**Multiple comparisons***
**DASS Stress Scale**	**I. No**	15.99	14.00	10.67	76.92	0.0125	0.0000	I vs. II (0.000)
	**II. Yes, I used psychological and psychiatric services**	23.29	24.00	11.62				I vs. III (0.000)
	**III. Yes, I used psychological services**	20.33	20.00	11.11				I vs. IV (0.001)
	**IV. Yes, I used psychiatric services**	20.70	21.00	11.02				

* only significant

Table S5: The results of the DASS total score for the economic situation during pandemics.

How do you assess your economic situation during pandemics?								
	**Group**	**Mean**	**Median**	**SD**	**Kruskal–Wallis H test**	**Bonferroni correction**	**p-value**	**Multiple comparisons***
**DASS Total Score**	**I. I have a stable family income, nothing has changed**	34.65	30.00	25.44	63.77	0.01	0.0000	I vs. IV (0.000)
	**II. I have a stable family income, but the situation is worse than before**	41.50	36.00	26.31				
	**III. I barely have enough money for living**	46.07	40.00	33.32				I vs. V (0.001)
	**IV. I have to start using savings**	46.20	40.00	28.34				
	**V. I have to borrow money from my family/friends during the outbreak of the pandemic because I do not have enough**	57.28	56.00	29.25				

	money to support myself							

* only significant

Table S5.2: The results of the DASS depression scale for the economic situation during pandemics.

How do you assess your economic situation during pandemics?								
	**Group**	**Mean**	**Median**	**SD**	**Kruskal–Wallis H test**	**Bonferroni correction**	**p-value**	**Multiple comparisons***
DASS Depression Scale	**I. I have a stable family income, nothing has changed**	12.36	10.00	10.09	51.87	0,01	0.0000	I vs. IV (0.000)
	**II. I have a stable family income, but the situation is worse than before**	14.36	12.00	10.24				
	**III. I barely have enough money for living**	18.77	16.00	14.63				I vs. V (0.001)
	**IV. I have to start using savings**	16.29	14.00	11.05				
	**V. I have to borrow money from my family/friends during the outbreak of the pandemic because I do not have enough money to support myself**	21.20	20.00	11.37				

* only significant

Table S5.3: The results of the DASS anxiety scale for the economic situation during pandemics.

How do you assess your economic situation during pandemics?								
	**Group**	**Mean**	**Median**	**SD**	**Kruskal–Wallis H test**	**Bonferroni correction**	**p-value**	**Multiple comparisons***
DASS Anxiety Scale	**I. I have a stable family income, nothing has changed**	6.67	4.00	7.81	58.80	0.01	0.000	I vs. IV (0.000)
	**II. I have a stable family income, but the situation**	8.86	6.00	8.53				

	**is worse than before**							
	**III. I barely have enough money for living**	9.03	8.00	7.52				
	**IV. I have to start using savings**	9.92	6.00	9.38				
	**V. I have to borrow money from my family/friends during the outbreak of the pandemic because I do not have enough money to support myself**	12.24	10.00	9.87				I vs. V (0.012)

* only significant

Table S5.4: The results of the DASS stress scale for the economic situation during pandemics.

**How do you assess your economic situation during pandemics?**								
	**Group**	**Mean**	**Median**	**SD**	**Kruskal–Wallis H test**	**Bonferroni correction**	**p-value**	**Multiple comparisons***
**DASS Stress Scale**	**I. I have a stable family income, nothing has changed**	15.62	14.00	10.72	51.44	0.01	0.0000	I vs. II (0.000)
	**II. I have a stable family income, but the situation is worse than before**	18.28	18.00	10.81				
	**III. I barely have enough money for living**	18.26	16.00	13.85				I vs. IV (0.000)
	**V. I have to borrow money from my family/friends during the outbreak of the pandemic because I do not have enough money to support myself**	23.84	28.00	12.36				I vs. V (0.006)

* only significant

Table S6: The results of the DASS total score for the current employment situation.

Are you currently working?								
	**Group**	**Mean**	**Median**	**SD**	**Kruskal–Wallis H test**	**Bonferroni correction**	**p-value**	**Multiple comparisons***
**DASS Total Score**	**I. No, I do not work**	39.11	34.00	26.66	17.76	0.0125	0.0005	I vs. II (0.012)
	**II. I work mentally**	34.09	28.00	26.65				
	**III. I work physically**	33.58	28.00	22.01				
	**IV. I run my own business**	26.29	19.00	23.10				

* only significant

Table S6.2: The results of the DASS depression scale for the current employment situation.

Are you currently working?								
	**Group**	**Mean**	**Median**	**SD**	**Kruskal–Wallis H test**	**Bonferroni correction**	**p-value**	**Multiple comparisons***
**DASS Depression Scale**	**I. No, I do not work**	13.92	12.00	10.47	30.50	0.0125	0.0000	I vs. II (0.002)
	**II. I work mentally**	11.69	8.00	10.27				
	**III. I work physically**	11.90	10.00	9.33				I vs. IV (0.000)
	**IV. I run my own business**	7.36	4.00	9.65				

* only significant

Table S6.3: The results of the DASS anxiety scale for the current employment situation.

Are you currently working?								
	**Group**	**Mean**	**Median**	**SD**	**Kruskal–Wallis H test**	**Bonferroni correction**	**p-value**	**Multiple comparisons***
**DASS Anxiety Scale**	**I. No, I do not work**	7.90	6.00	8.42	6.33	-	0.0967	-
	**II. I work mentally**	6.91	4.00	7.95				
	**III. I work physically**	6.92	4.00	7.16				
	**IV. I run my own business**	5.07	2.00	6.05				

* only significant

Table S6.4: The results of the DASS stress scale for the current employment situation.

Are you currently working?

	Group	Mean	Median	SD	Kruskal–Wallis H test	Bonferroni correction	p-value	Multiple comparisons*
DASS Stress Score	**I. No, I do not work**	17.29	16.00	10.92	13.96	0.0125	0.0030	-
	**II. I work mentally**	15.49	14.00	11.47				
	**III. I work physically**	14.77	14.00	9.94				
	**IV. I run my own business**	13.86	11.00	11.59				

* only significant

Table S7: The results of the DASS total score for the current living situation.

I live with:								
	**Group**	**Mean**	**Median**	**SD**	**Kruskal–Wallis H test**	**Bonferroni correction**	**p-value**	**Multiple comparisons***
**DASS Total Score**	**I. Alone**	34.96	30.00	26.42	16.37	0.01	0.0026	II vs. III (0.015)
	**II. Parents**	49.53	34.00	27.02				
	**III. Partner or spouse**	34.35	26.00	36.11				
	**IV. Roommates**	39.03	34.00	25.82				
	**V. Partner/spouse and children**	30.95	28.00	19.11				

* only significant

Table S7.2: The results of the DASS depression scale for the current living situation.

I live with:								
	**Group**	**Mean**	**Median**	**SD**	**Kruskal–Wallis H test**	**Bonferroni correction**	**p-value**	**Multiple comparisons***
**DASS Depression Scale**	**I. Alone**	13.07	10.00	10.63	37.23	0.01	0.0000	II vs. III (0.000)
	**II. Parents**	14.24	12.00	10.59				
	**III. Partner or spouse**	10.79	8.00	9.65				
	**IV. Roommates**	13.88	12.00	10.32				
	**V. Partner/spouse and children**	8.48	8.00	7.67				

* only significant

Table S7.3: The results of the DASS anxiety scale for the current living situation.

I live with:								
**DASS Anxiety Scale**	**Group**	**Mean**	**Median**	**SD**	**Kruskal–Wallis H test**	**Bonferroni correction**	**p-value**	**Multiple comparisons***

	**I. Alone**	7.03	4.00	7.88	5.86	-	0.2102	-
	**II. Parents**	7.96	6.00	8.43				
	**III. Partner or spouse**	7.34	4.00	8.50				
	**IV. Roommates**	7.80	6.00	8.18				
	**V. Partner/spouse and children**	5.05	4.00	5.05				

* only significant

Table S7.4: The results of the DASS stress scale for the current living situation.

**I live with:**								
	**Group**	**Mean**	**Median**	**SD**	**Kruskal–Wallis H test**	**Bonferroni correction**	**p-value**	**Multiple comparisons***
**DASS Stress Scale**	**I. Alone**	14.87	14.00	11.31	13.12	0.01	0.0107	I vs. II (0.012)
	**II. Parents**	17.33	16.00	11.09				
	**III. Partner or spouse**	16.22	14.00	10.85				I vs. IV (0.019)
	**IV. Roommates**	17.36	16.00	10.69				
	**V. Partner/spouse and children**	17.43	16.00	9.10				

* only significant

Table S8: The sociodemographic data of the respondents.

**SOCIODEMOGRAPHIC DATA**								
Variables		DASS 0		DASS 2		OR	IC	s
		**n**	**%**	**n**	**%**			
Sex (p<0.00001)	Women	198	60%	316	82%	3.0121	2.1479 to 4.2239	0.0001
	Men	134	40%	71	18%			
Field of study (p=0.02778)	Arts and humanities	10	3%	23	6%	1.9826	0.9301 to 4.2258	0.07
	Sciences	11	3%	26	7%	2.0374	0.9916 to 4.1863	0.05
	Medicine	209	63%	206	54%	0.8496	0.6669 to 1.0824	0.187
	Engineering	32	10%	38	10%	1.0236	0.6254 to 1.6754	0.926
	Social sciences	69	21%	91	24%	1.1368	0.8046 to 1.6063	0.467
I live with: (p=0.05078)	Alone	49	15%	35	9%	0.6128	0.3877 to 0.9686	0.03
	Parents	157	47%	206	53%	1.1256	0.8730 to 1.4514	0.361
	Roommates	65	20%	95	25%	1.2538	0.8854 to 1.7755	0.202
	Partner or spouse	60	18%	49	13%	0.7006	0.4673 to 1.0503	0.08
	Partner/spouse and children	1	0%	1	0%	0.8579	0.0534 to 13.7693	0.913
**DATA RELATED TO HEALTH**								
Do you have any chronic disease?	No	306	92%	295	76%			
	Thyroid diseases	7	2%	25	6%	3.7046	1.5783 to 8.6957	0.002

(p<0.00001)	Mental disorders	3	1%	17	4%	5.8780	1.7048 to 20.2668	0.005
	Other	8	2%	26	7%	3.3712	1.5021 to 7.5661	0.003
Did you use psychological/psychiatric help before the beginning of the pandemics? (p<0.00001)	No	304	92%	264	68%			
	Yes, I used psychological services	16	5%	44	11%	3.1667	1.7458 to 5.7440	0.0001
	Yes, I used psychiatric services	4	1%	28	7%	8.0606	2.7911 to 23.2789	0.0001
	Yes, I used psychological and psychiatric services	8	2%	51	13%	7.3409	3.4216 to 15.7497	0.0001
Did you use psychological/psychiatric help during the pandemics? (P<0.00001)	No	330	99%	352	91%			
	Yes, I used psychological services	2	1%	13	3%	0.1442	0.0323 to 0.6440	0.01
	Yes, I used psychiatric services	0	0%	12	3%	-		
	Yes, I used psychological and psychiatric services	0	0%	10	3%	-		
What was the most difficult for you during pandemics? (p<0.00001)	Fear of being infected	11	3%	17	4%	62.3913	3.4246 to 1136.6806	0.005
	Fear of infection of the loved ones	106	32%	105	27%	41.3886	2.4711 to 693.2239	0.009
	Concern about education	12	4%	8	2%	27.8800	1.4773 to 526.1439	0.02
	Loneliness	6	2%	42	12%	293.3077	15.7710 to 5454.9184	0.0001
	Isolation	26	8%	43	11%	67.3019	3.9058 to 1159.7023	0.003
	Financial unstability	30	9%	32	8%	43.6885	2.5306 to 754.2515	0.009
	Change of the lifestyle	41	12%	70	18%	69.6506	4.1039 to 1182.0880	0.003
	Changes awaiting the world after the pandemics	79	24%	65	17%	33.7799	2.0045 to 569.2524	0.01
	I was not afraid	20	6%	0	0%			
**ECONOMIC SITUATION**								
Are you currently working? (p=0.00191)	No, I do not work	258	78%	340	88%			
	I work mentally	52	16%	37	10%	0.5399	0.3437 to 0.8481	0.007
	I work physically	14	4%	8	2%	0.4336	0.1792 to 1.0492	0.06
How do you assess your economic situation during pandemics? (p<0.00001)	I have a stable family income, nothing has changed	236	71%	175	45%	-	-	-
	I have a stable family income,	73	22%	135	35%	2.4939	1.7655 to 3.5229	0.0001

	but the situation is worse than before							
	I have to start using savings	15	5%	57	15%	5.1246	2.8084 to 9.3511	0.0001
	I have to borrow money from my family/friends during the outbreak of the pandemic because I do not have enough money to support myself	1	0%	10	3%	13.4857	1.7103 to 106.3328	0.01
	I barely have enough money for living	4	1%	10	3%	1.9265	0.7191 to 5.1614	0.192
